# Comparative evaluation of antifibrotic activity of *Salvadora persica* extracts in CCl4-induced liver fibrosis in rats by reducing oxidative stress and inflammation via transcriptional modulation associated with TGF-β1/Smad signaling pathway

**DOI:** 10.3389/fphar.2026.1900981

**Published:** 2026-10-08

**Authors:** Fouzia Latif, Bilal Ahmed, Hamad Z. Alkhathlan, Muhammad Saad Majeed, Afia Muhammad Akram, Qurat-ul-ain Ahmad, Shehla Akbar, Sabira Sultana, Hailing Ding, Farhan Sachal Cyprian, Merajuddin khan

**Affiliations:** 1 Faculty of Medicine, Qilu Institute of Technology, Jinan, Shandong, China; 2 Department of Chemistry, College of Science, King Saud University, Riyadh, Saudi Arabia; 3 Faculty of Medicine and Allied Health Sciences, The Islamia University of Bahawalpur, Bahawalpur, Pakistan; 4 Department of Zoology, Division of Science and Technology, University of Education, Lahore, Pakistan; 5 College of Pharmacy, University of Sargodha, Sargodha, Pakistan; 6 Department of Eastern Medicine, Government College University Faisalabad, Faisalabad, Pakistan; 7 College of Medicine, Qatar University, Doha, Qatar

**Keywords:** anti-inflammatory, antioxidants, collagen deposition, liver fibrosis, Salvadora persica extract

## Abstract

Plant *Salvadora persica* (SP) is well-known for its pharmacological activities, including its role in oral hygiene. In the present study, we investigated, the *in vivo* comparative hepatoprotective potential of SP root extracts against chronic liver injury fibrosis. In Wistar albino rats, liver fibrosis was induced by intraperitoneal injection of carbon tetrachloride (CCl4) in olive oil (1:1 v/v) at 1 mL/kg, twice weekly for 9 weeks. Liver damage was evaluated by measuring serum liver biochemical parameters, antioxidant enzyme levels, and lipid peroxidation marker in liver homogenate. The mRNA expression level of inflammatory and profibrotic markers in liver tissue and histopathology of liver tissue by H&E staining and collagen deposition by Masson’s trichrome, and Van Gieson’s staining were determined. SP extracts, including aqueous (SP-AQ), ethanolic (SP-E), and petroleum ether (SP-PE), were analyzed by Gas Chromatography-Mass Spectrometry (GC-MS), followed by multivariate principal component analysis (PCA) and partial least squares discriminant analysis (PLS-DA). The findings demonstrated that SP extracts demonstrated significant hepatoprotective effects by improving rats’ body weight without significant increase in liver index, reducing liver biochemical markers, and reducing oxidative stress by increasing SOD and GPx levels and decreasing MDA level. In liver tissue, the mRNA expression levels of the inflammatory markers IL-1β, IL-6, and TNF-α and profibrotic markers TGF-β1, Smad2, Smad3, α-SMA, and Collagen1A1 were downregulated by modulating the genes expression levels related with the TGF-β1/Smad signaling pathway. Histopathology showed recovery from liver damage and reduced collagen deposition. Comparatively, antifibrotic activity was greater by SP-PE, modest by SP-E and lowest by SP-AQ extracts. Furthermore, phytochemical analysis of the SP extracts demonstrated considerable diversity in their phytoconstituent profiles due to difference in solvent polarity, with PCA and PLS-DA analyses confirming distinct clustering and discrimination among SP extracts. SP-PE extract, greater efficacy can be attributed to selection of a non-polar solvent and 4-propenyl-2,6-dimethoxyphenol, along with other dominant phytochemicals, which exerted synergistic biological effects. The SP extracts showed significant hepatoprotective activity with consistent biological effects in reducing oxidative stress, inflammation, and fibrosis in CCl4-induced liver fibrosis in rats. However, further studies of major individual compounds of SP-PE are warranted for clinical use in liver fibrosis.

## 1 Introduction

Liver fibrosis is a chronic liver injury that is a major global health issue due to reported mortality and morbidity at a significant level, and the global prevalence rate of advanced liver fibrosis is 3.3% (Zamani et al., 2024). Liver fibrosis is characterized by excessive synthesis and accumulation of extracellular matrix (ECM) proteins and disruption of the architecture of the normal liver. Etiological factors involving reactive oxygen species (ROS) induced high inflammatory conditions; hepatitis B, hepatitis C, intake of excessive alcohol, and metabolic ailments, develop fibrosis, cirrhotic liver, and liver cancer (Parola and Pinzani, 2024). In the normal liver, the Disse space extracellular matrix (ECM) is composed of glycoproteins, proteoglycans, and non-fibrogenic type IV collagen, which provide mechanical support and molecular signals for liver cell function. But when liver injury occurs, the ECM density and composition change, and ECM production increases by about 6-8-fold. During liver injury, the non-fibrogenic component type IV collagen of the ECM is replaced by fibrogenic components, types I, II, and III collagen (Acharya et al., 2021; Garbuzenko, 2022). In fibrosis, activation of hepatic stellate cells (HSCs) causes quiescent vitamin A storing cells to transition into fibrogenic myofibroblasts that produce and release excessive amounts of fibrillar collagen types I and III, components of the ECM (Garbuzenko, 2022). During liver fibrosis, hepatocyte death by apoptosis or necrosis triggers inflammation in the liver, resulting in the recruitment of inflammatory cells, including Kupffer cells (KCs), which are the resident liver macrophages (Dawood et al., 2020). Engulfment of apoptotic bodies of hepatocytes by KCs initiates and promotes hepatic inflammation leading to fibrosis, which is facilitated by cytokines, interleukin-6 (IL-6), tumor necrosis factor-alpha (TNF-α) and transforming growth factor-beta1 (TGF-β1) (Hammerich and Tacke, 2023; Das et al., 2026a). The cytokine TGF-β1 is a profibrotic mediator that is crucial for the activation of quiescent HSCs, which then produce excessive ECM, leading to liver fibrosis (Dewidar et al., 2019). Improper treatment of fibrosis can progress to liver cirrhosis, liver cancer, and death. Synthetic drugs used in treating liver diseases have evidenced life-threatening conditions, thus preference is shifting to natural products, which have shown their efficacy in liver fibrosis treatment (Ciceu et al., 2024). Plant *Salvadora persica* (SP), also called toothbrush tree (miswak), belonging to the Salvadoraaceae family, is found in tropical and subtropical Asia. SP is a small tree or multibranch shrub with a height of 6-7 m. The branches are droopy and the bark is rough. The leaves are circular, the flowers are small with a fruit cluster (Kumar and Sharma, 2021). SP has demonstrated many pharmacological effects, including antioxidant, anti-inflammatory, antimicrobial, antiosteoporosis, wound-healing, antiulcer, diuretic, analgesic, astringent, anthelmintic, antidiabetic, antihyperlipidemic, anticonvulsant, sedative and anticancer activities (Farag et al., 2021). SP is a natural food preservative and also used as a food ingredient (Kumari et al., 2017). The fruits, leaves, seeds, and root bark exert purgative activity and are also used to treat fever, cough, and asthma. The roots are also used to treat various chest diseases. The leaves and young branches, being rich in water (15%-36%), are suitable fodder for camels (Korejo et al., 2010). Phytochemical analysis of SP shows the presence of alkaloids, terpenes, glycosides, flavonoids, phenolic acids, carbohydrates, saponins, isothiocyanates, sterols, fatty acids, calcium, fluoride, vitamin C and chloride (Akhtar et al., 2011; Alaraj et al., 2021). The non-targeted Gas Chromatography-Mass Spectrometry (GC-MS), is one of the most efficient methods for simultaneously detecting a wide variety of phytochemicals in plant extracts (Sasse and Rainer, 2022). In several previously reported studies, the SP acetone, aqueous, and alcoholic root extracts have demonstrated antimicrobial and antioxidant activities (Khan et al., 2020; Aljarbou et al., 2022). In previous research, the ethanolic extract of SP roots has shown anti-inflammatory activity by reducing paw edema volume (Hoor et al., 2014). The SP root aqueous extract exhibited antioxidant and hepatoprotective effects against paracetamol-induced toxicity in rats, as evidenced by decreased serum liver biochemical parameters (Qureshi et al., 2016)]. In another study, the SP aqueous extract of the root part showed protective activity against paracetamol-induced hepatorenal toxicity by reducing liver biochemical markers and improving histopathological recovery of damaged liver and kidney tissue, as assessed by H&E staining (Alaraj et al., 2021). The SP aqueous extract of the root part has shown hepatorenal protective activity in cyclophosphamide-induced hepatorenal toxicity in rats by showing the decrease in elevated serum toxicity biochemical markers, alleviating inflammatory mediators levels, and increasing the antioxidant enzyme levels in liver tissues, histopathological restoration of hepatorenal damage and reducing the apoptosis (Bokhary et al., 2022). Previously reported literature provides experimental evidence of hepatoprotective activity of SP root aqueous extract against acute liver damage, attributed to its antioxidant and anti-inflammatory effects. To date, no study on SP has reported its *in vivo* hepatoprotective potential against chronic liver injury, including CCl4-induced liver fibrosis, nor evaluated collagen deposition, alterations in body and liver weight, or the effects of SP extract treatment on the mRNA expression levels of profibrotic markers in rat liver tissue. Furthermore, comparative evaluation of the SP various extracts for their efficacy in reversing liver damage has not yet been investigated. Therefore, the present study was designed to evaluate the *in vivo* hepatoprotective activity of various SP extracts against CCl4-induced liver fibrosis in rats. In histopathology, fibrosis-specific staining, including Masson’s trichrome and Van Gieson’s stains, was employed to assess collagen deposition in liver tissue. In addition, rats’ body weight, liver index, liver biochemical parameters, antioxidant enzymes, lipid peroxidation level, and mRNA expression of inflammatory and profibrotic markers by modulating the genes associated with modulation of TGF-β1/Smad signaling pathway in liver tissue were analyzed. Moreover, the characteristic phytochemical profile of SP extracts was comprehensively investigated by GC–MS, and discrimination among SP extracts was assessed using multivariate principal component analysis (PCA) and partial least squares discriminant analysis (PLS-DA). Our study provides new insights into the effectiveness and therapeutic applications of SP extracts by decreasing oxidative stress and inflammation with a molecular mechanism in liver fibrosis.

## 2 Materials and methods

### 2.1 Collection of plant materials

The SP root sticks were purchased from a local herbal supplier on Raiwind Road, Lahore, Pakistan. These plant samples were identified and verified by Professor Xu Nan from the Shandong Research Academy of Traditional Chinese Medicine (Jinan, China). A voucher number (3953) was deposited at the herbarium of the Medical Department.

### 2.2 Plant extracts preparation

Authenticated SP root sticks were cut into small pieces and air dried in shade at room temperature. The dried plant materials were ground into a powder and extracted by maceration using solvents of different polarities, such as aqueous solution, petroleum ether and ethanol. Each 100 g of stick powder was separately soaked in 1 L of each of these solvents for 3 days with occasional shaking. Then, filtration was carried out using Whatman # 1 filter paper in a flask, followed by evaporation by rotary evaporator under 40 °C for further utilization. The extraction yield of the extract was calculated using the following formula.

Extraction Yield (%)=Weight of dried extract/Weight of plant material×100

The % yield of the SP-AQ, SP-E and SP-PE extracts was 2.7 g, 2.4 g and 1.8 g. The complete steps of plant extract preparation are given below in a flow diagram (Figure 1).

**FIGURE 1:**
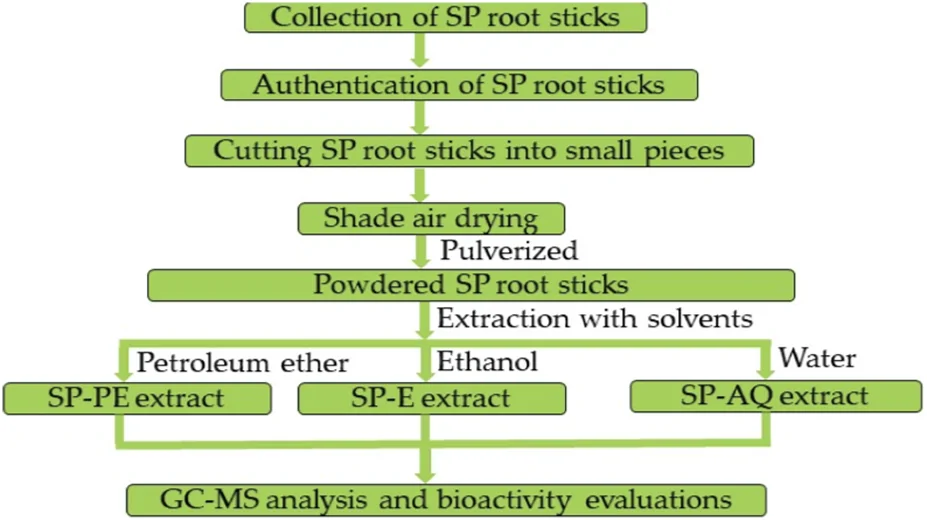
Flow diagram of SP extracts preparation.

### 2.3 Animals handling for *in vivo* study

The *in vivo* rat experiments of this study were approved by the Animal Ethics Committee of Qilu Institute of Technology, Jinan, Shandong, China (Approval number: 3701810047642; Date: 27 May 2025). All experiments were performed according to Guide for Animal care and Use. Forty-eight Wistar albino rats of either sex with an average body weight (b.w.) of 190–198 g of 7-8 weeks were obtained from Qilu Institute of Technology, Jinan, China. Rats were kept under standard conditions of a 12 h light/dark cycle, at 24 °C ± 1 °C and 55% ± 5% humidity, with free access to water and food. Rats were consumed for the treatment protocol after 1 week of acclimatized conditions under standard laboratory conditions. Figure 2 illustrates the overall experimental design for the animal study.

**FIGURE 2:**
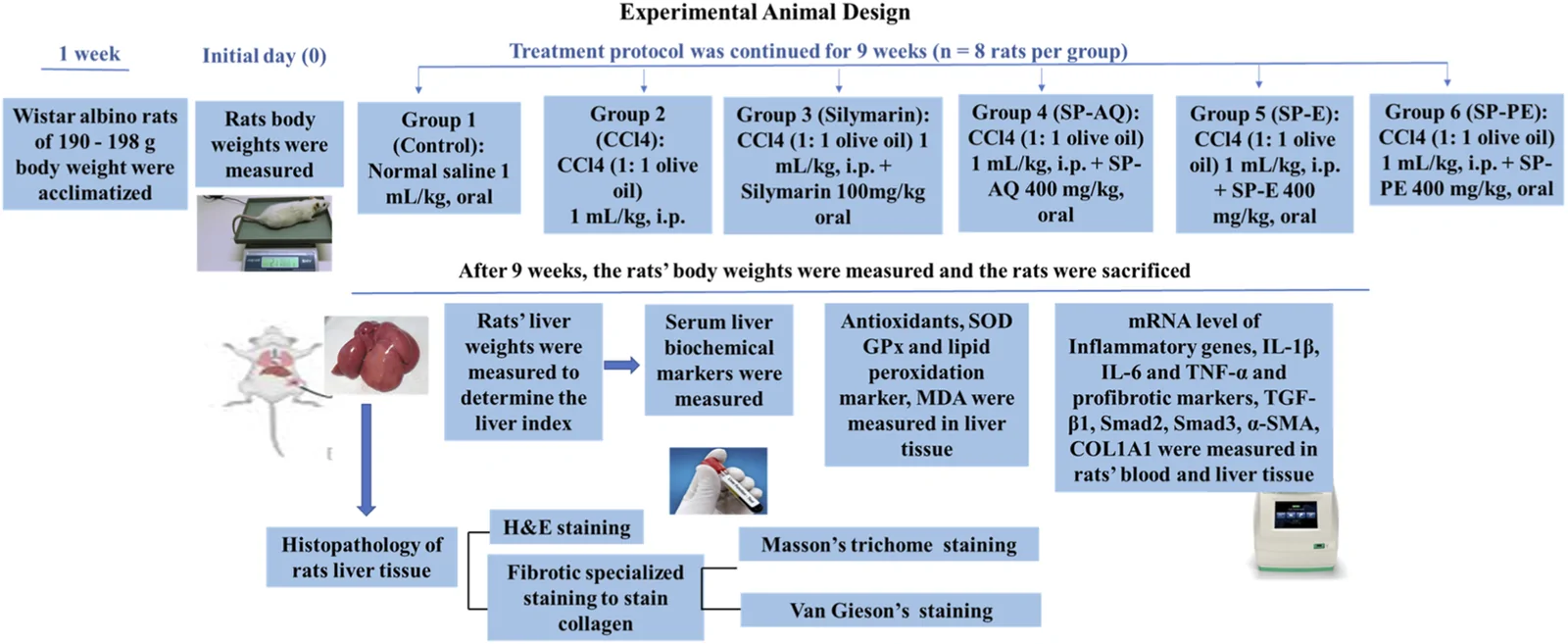
Outline of experimental animal study design.

#### 2.3.1 Treatment protocol

Wistar albino rats (n = 48) were randomly divided into six groups (8 rats/group) based on body weight of 190-198 g. In rats, liver damage was induced by CCl4 diluted in olive oil at a 1: 1 (*v/v*) ratio. This CCl4 solution (1 mL/kg) was used in the experimental study to induce fibrosis in rats. The Treatment protocol was followed as described by (Rahmani et al., 2024), with slight modifications: the protocol was extended to 9 weeks, and the drug silymarin (Legalon®, Viatris, China) (Olusanya, 2025) was used in the standard rat group. The formulations of SP extracts and silymarin were prepared separately in normal saline as stock suspensions, without using a suspending agent, mixed by vortexing to ensure homogeneity and uniformity of formulations, and the volume to be administered orally was measured immediately based on each rat’s body weight. The fresh dosing for all experimental groups was prepared and administered in the same way throughout the 9 weeks study.

Different treatment protocols were given to the rat groups for 9 weeks.

Group I: (Control group): rats were fed orally with normal saline at 1 mL/kg, b.w. twice a week.

Group II: (CCl4 group): rats were injected intraperitoneally (i.p.) with CCl4 at 1 mL/kg, b.w. twice a week.

Group III: (silymarin fed group): rats were fed orally with 100 mg/kg, b.w. of the standard drug, silymarin, twice a week and injected i.p. with CCl4 at 1 mL/kg, b.w. twice a week.

Group IV: (SP-AQ fed group): rats were fed orally with 400 mg/kg, b.w. of SP-AQ extract twice a week and injected i.p. with CCl4 at 1 mL/kg, b.w. twice a week.

Group V: (SP-E fed group): rats were fed orally with 400 mg/kg, b.w. of SP-E extract twice a week and injected i.p. with CCl4 at 1 mL/kg, b.w. twice a week.

Group VI: (SP-PE fed group): rats were fed orally with 400 mg/kg, b.w. of SP-PE extract twice a week and injected i.p. CCl4 at 1 mL/kg, b.w. twice a week.

#### 2.3.2 Rat body weight and liver weight

At the end of 9 weeks, the rats’ body weight was measured. Rats were kept fastened overnight, then rats were sacrificed after being given general anesthesia using ketamine and xylazine. Rats’ livers were weighed and washed with normal saline to remove blood and the liver index was calculated using the following formula.

Liver index = rat liver weight/rat body weight × 100%

#### 2.3.3 Rat blood collection

After 9 weeks rats were given short-term anesthesia with isoflurane to minimize anesthesia-induced alterations in liver biochemical parameters. The blood was collected immediately by intracardiac puncture into ethylenediaminetetraacetic acid (EDTA) tubes for RT-qPCR analysis and into serum-separating tubes to obtain the supernatant for liver biochemical analysis. Subsequently, rats were given under deep anesthesia with ketamine and xylazine, followed by euthanasia according to institutional standard protocol and the liver tissues were harvested.

#### 2.3.4 Measurement of rat serum biomarkers of liver function

Rat blood samples were centrifuged at 3000 *g* for 15 min and serum was used for measurement of liver function markers including liver enzymes; aspartate aminotransaminase (AST), alanine aminotransaminase (ALT), alkaline phosphatase (ALP), liver excretory function marker; Total bilirubin and liver protein synthesis markers; Total protein, albumin, and globulin using commercial detection kits according to manufacturer’s described protocol (Nanjing Jiancheng Biological Engineering Institute, Jiangsu, China).

#### 2.3.5 Measurement of oxidative stress markers in rat liver tissue

Some of the rat liver tissue, 100 mg was homogenized in an ice-cold 100 mM KH2PO4 buffer containing 1 mM ethylenediaminetetraacetic acid (EDTA), pH 7.4, to obtain 10% homogenate (1:9 w/v) and then centrifuged at 120,00 *g* for 20-25 min at 4 °C. The supernatant was collected to assess antioxidant enzymes, superoxide dismutase (SOD), glutathione peroxidase (GPx), and lipid peroxidation marker, malondialdehyde (MDA), using commercial detection kits according to the manufacturers’ protocols (Nanjing Jiancheng Biological Engineering Institute, Jiangsu, China).

#### 2.3.6 RNA extraction from rat blood and liver tissue for RT-qPCR assay

Rat liver was dissolved in −196 °C liquid nitrogen, and the powder was prepared for use in RNA extraction. RNA was extracted from biological samples, homogenized liver and rat blood using TRIzol (Invitrogen, CA, United States) and the measurement of isolated RNA concentration was performed using Nanodrop (Applied Biosciences, CA, United States). The cDNA was prepared according to the manufacturer’s protocol (Thermo Fisher Scientific, MA, United States). The 10 µL reaction mixture was prepared by adding 0.2 µL forward primer, 0.2 µL reverse primer, 5 µL MIX, 2 µL cDNA and deionized water. The RT-qPCR assay using LUX™ primers of inflammatory markers, interleukin-1 beta (IL-1β), IL-6, TNF-α and profibrotic marker, TGF-β1 was performed by thermocycler (qTower^3^, Real-time PCR, Analytic Jena AG, Jena, Germany) and cycling condition was followed by manufacturer of TaqMan Master Mix Kit (Thermo Fisher, Waltham, MA, United States) by keeping β-actin as internal control. Gene expression was calculated using the Ct (2^−ΔΔCt^) method. The sequence of LUX™ primers of inflammatory and profibrotic genes were designed using IDT software (Table 1) and LUX™ primers were synthesized by ShineGene Biotechnology, Shanghai, China.

**TABLE 1:** Primer sequence of inflammatory and profibrotic genes.

LUX^TM^ primers	F (5’-3’) R 3’-5’)	Primer sequence	Amplicon size	Fluorochrome dye
IL-6	F R	CAA AGC GGG TCT AGA CAT GTT CAG CTT TG GGT TTC CAC GTC TCA CCA TTA G	157	3-JOE
IL-1β	F R	TGA AGG AGA TGT CCA CCT TCA CCT TCA CTG GGT TGG ATG GTC TCT TCTA	162	3-FITC
TNF-α	F R	GAT AGCACG GAG CTA AACTAC CAG CTA TC CCA CAT CTC GGA TCA TGCTTT C	144	3-CY3
TGF-β1	F R	TCT ACG ACT GTG GAG CAA CAC GTA GACTG AAG CGA AAG CCCTGT ATT C	147	3-FITC
Smad2	F R	CT AGC ACT GTG GCG CAA CAC GCA GA TATCGTGA ATCCAGG TCGCCTG	138	3-JOE
Smad3	F R	GCAAGATTGGCTACCTGGCAA CCGGCCACAAGTGGACCGGT	145	3-JOE
α-SMA	F R	TAACCCAAAGCAGAGTTAGC AGTTTGGTTCTCATTATTC	152	3-CY3
COL1A1	F R	GAGTCTTGGAGGTTTCATGGATTCG AGTCTGACCTTTCATCCAGG	243	3-FITC

#### 2.3.7 Histopathological evaluation

Rat liver tissues fixed in 10% neutral buffered formalin, embedded in paraffin, were cut into 5 µm thick sections. These tissue sections were stained with hematoxylin and eosin (H&E) to observe the morphology of the overall diseased liver area. The fibrotic specific stains, including Masson’s trichome stain (Thermo Fisher, Waltham, MA, United States), to primarily stain the total collagen mainly, type I collagen and type III collagen, and Van Gieson’s stain (Thermo Fisher, Waltham, MA, United States), to primarily stain the type I collagen (Guo et al., 2015) to assess the fibrotic area of liver tissue and collagen accumulation under microscopy examination.

### 2.4 GC-MS analysis of various SP extracts

The GC-MS analysis was conducted using a TRACE 1610 GC chromatograph, coupled with an Orbitrap Exploris mass spectrometry (Thermo Fischer Scientific, United States) at Majorbio Bio-Pharm Technology Co. Ltd. (Shanghai, China). The instrument utilized an inert electron impact (EI) ionization source, operated at 70 eV (Agilent, United States). The sample separation was performed using a TG-5SLIMS (30 × 0.25 mm × 0.25 µm) capillary column with 99.99% helium as the carrier gas at a constant flow rate of 1 mL/min. The 1 µL of sample was injected in split mode (10: 1) at an inlet temperature of 300 °C. The GC column temperature was initially held at 80 °C for 0 s, then raised to 310 °C at 20 °C/min for 8 min, with a total run time of 20 min. The mass spectrometry performed analysis at an ion source temperature of 250 °C in full scan mode, m/z 35-500 with a resolving power of full width at half maximum. The alkane standards series (C10-C33) was analyzed under the same chromatographic conditions for calculating RI.

#### 2.4.1 Phytoconstituents identification of various SP extracts

Phytoconstituents present in the various SP extracts were identified using complementary parameters (i) mass spectral data and retention indices (RIs) by comparison with the NIST 2023 library, the Thermo Scientific GC-Orbitrap Metabolism v2.0 library, and a self-constructed library developed by Majorbio, (ii) comparison of experimental and literature retention indices, and (iii) agreement with previously published reports on SP phytochemistry when available. Results are reported according to their elution order on the TG-5SLIMS column.

### 2.5 Multivariate PCA and PLS-DA analysis

PLS-DA multivariate analysis was performed to determine the extracts separation. For this purpose, the GC-MS data matrix of peak areas (intensities) was uploaded to the Majorbio Cloud platform for further analysis. The errors arising from instrument instability and sample preparation were minimized by normalizing the response intensities of mass spectrometry peaks of the samples using the sum normalization method, resulting in a normalized data matrix. The data were log10-transformed to generate a final data matrix for subsequent sample analysis. For multivariate statistical analysis, the R package “ropls” version 1.6.2 was used to perform principal component analysis (PCA) and PLS-DA. Model robustness was assessed using seven-fold cross-validation. Based on the variable importance in projection (VIP) scores obtained from PLS-DA, metabolites were considered significantly different if VIP >1 and p-value <0.05, and the p-value was calculated using Student’s t-test.

### 2.6 Data analysis

The experimental data (n = 8) were analyzed using GraphPad Prism 9.0.0 (GraphPad Software, MA, United States). One-way ANOVA with Bonferroni test was applied to provide a conservative assessment of statistical significance among different experimental groups. The results were expressed as mean ± SEM with a *p* < 0.05 and 95% CI, indicating statistically significant value.

## 3 Results

### 3.1 SP extracts effect on rat body weight and liver weight

Rats’ body weight was measured on the initial (0) day and at the end of 9 weeks, and liver weight was measured after sacrifice at the end of 9 weeks. During the 9 weeks study, we did not observe any apparent signs of toxicity or mortality in rats. The CCl4 group showed a highly significant difference in body weight (*p* < 0.0001) compared with the control group. Compared to the CCl4 group, in the SP extracts treated rats, the increase in body weight was non-significant by SP-AQ extract, moderate (*p <* 0.001) by SP-E and pronounced (*p <* 0.0001) by SP-PE. The silymarin group showed the most significant increase (*p* < 0.0001) in body weight compared with the CCl4 group, which was close to the control group (Table 1). Compared to the control group, the liver weight and liver index of the CCl4-intoxicated rats were increased most significantly (*p <* 0.0001). Compared to CCl4 group, the SP-AQ extract showed a marginal decrease (*p <* 0.05), SP-E, a modest decrease (*p <* 0.001) and SP-PE, a pronounced decrease (*p <* 0.0001) in rats’ liver weight and liver index (Table 2). SP extracts improved body weight, and the liver index was not increased, compared with the CCL4 group, most effectively with the SP-PE extract.

**TABLE 2:** The effect of SP extracts on rats’ body weight and liver weight.

Groups	Body weight (g)		Liver weight (g) at day 63	Liver index
	Initial (0) day	At day 63		
Control	190.83 ± 4.64	234.33 ± 4.24	7.76 ± 0.23	3.3 ± 0.02
CCl4	190.33 ± 6.50	201.83 ± 3.34^####^	10.45 ± 0.38	5.1 ± 0.05^####^
Silymarin	191.00 ± 9.0	229.50 ± 2.03****	8.21 ± 0.40	3.5 ± 0.08****
SP-AQ	193.50 ± 5.18	211.67 ± 6.24 ^ns^	9.14 ± 0.61	4.3 ± 0.02*
SP-E	198.50 ± 6.77	225.50 ± 6.94***	8.93 ± 0.29	3.9 ± 0.13***
SP-PE	194.50 ± 7.22	227.16 ± 8.00****	8.42 ± 0.68	3.7 ± 0.01****

Data was presented as mean ± SEM (*n* = 8). One-way ANOVA, followed by the Bonferroni test, was applied for statistical significance.

^#^ ^###^ *p* < 0.0001 compared to control group; ns, non-significant, *p < 0.05, ***p < 0.001; ****p < 0.0001, compared to CCl4 group.

### 3.2 Attenuation in liver biochemical parameters

The extent of hepatocyte damage was assessed by measuring serum liver biochemical parameters ALT, AST, ALP, and Total bilirubin, and increased the Total protein, albumin, and globulin levels in rats. The CCl4 group showed a highly significant increase (*p* < 0.0001) in liver biochemical markers compared to control group. SP extracts reduced the elevated liver biochemical markers compared to the CCl4 group; SP-AQ extract slightly reduced (*p* < 0.01), SP-E and SP-PE extracts remarkably reduced (*p* < 0.0001). Silymarin most effectively reduced (*p* < 0.0001) elevated levels of biochemical markers, which were comparable to those in the control group (Table 3).

**TABLE 3:** SP extracts effect on serum liver biochemical parameters.

Groups	ALT (IU/L)	AST (IU/L)	ALP (IU/L)	Total bilirubin (mg/dL)	Total protein (g/dL)	Albumin (g/dL)	Globulin (g/dL)
Control	51.160 ± 0.60	97.47 ± 2.44	148.00 ± 0.37	0.38 ± 0.01	5.95 ± 0.37	3.52 ± 0.01	2.63 ± 0.01
CCl4	96.00 ± 1.63^####^	246.26 ± 1.33^####^	475.42 ± 0.87^####^	1.29 ± 0.09^####^	2.27 ± 0.13^####^	1.52 ± 0.02^###^	0.42 ± 0.09^###^
Silymarin	64.15 ± 1.45****	114.66 ± 1.91****	167.53 ± 1.33****	0.46 ± 0.06****	5.72 ± 0.01****	3.32 ± 0.03****	2.25 ± 0.06****
SP-AQ	84.36 ± 1.49**	199.83 ± 2.25***	361.50 ± 2.14***	0.87 ± 0.07***	3.26 ± 0.01***	1.89 ± 0.07***	1.26 ± 0.01***
SP-E	79.68 ± 1.68****	152.16 ± 0.27****	232.66 ± 1.45****	0.72 ± 0.14****	4.83 ± 0.05****	2.41 ± 0.06****	1.73 ± 0.02****
SP-PE	71.24 ± 1.14****	131.78 ± 1.04****	186.72 ± 1.94****	0.59 ± 1.02****	5.24 ± 0.03****	3.07 ± 0.02****	1.94 ± 0.02****

Data were expressed in mean ± SEM (*n* = 8). One-way ANOVA, was applied, followed by the Bonferroni test for statistical analysis.

^#^ ^###^ *p* < 0.0001 compared with control group; **p < 0.01, ***p < 0.001, ****p < 0.001 compared with CCl4 group.

### 3.3 SP extracts effect on oxidative stress markers

The antioxidative enzymes, SOD and GPx and lipid peroxidation marker, MDA were measured in rat liver tissue. Severe hepatotoxicity was demonstrated in the CCl4 group (*p* < 0.0001) compared to the control group. Compared to the CCl4 group, the recovery from hepatotoxicity, as evidenced by decreased MDA levels and increased SOD and GPx levels, was observed less with SP-AQ extract (*p* < 0.001), modest with SP-E extract and most considerable with SP-PE extract (*p* < 0.0001) (Table 4). The silymarin group showed a highly significant increase in SOD and GPx activities and a reduction in MDA levels (*p* < 0.0001) compared with the CCl4 group, which was close to the control group.

**TABLE 4:** Antioxidant activity of SP extracts in rat liver tissue.

Groups	SOD (U/mg protein)	GPx (nmol/mg protein)	MDA (nmol/gm tissue)
Control	8.11 ± 0.23	98.27 ± 0.05	1.76 ± 1.21
CCl4	4.64 ± 0.71^####^	44.25 ± 0.62^####^	6.71 ± 0.95^####^
Silymarin	7.64 ± 0.55****	87.12 ± 0.14****	2.64 ± 0.47****
SP-AQ	5.42 ± 0.62***	58.44 ± 0.07***	5.36 ± 0.08***
SP-E	6.63 ± 0.58****	73.31 ± 1.16****	4.58 ± 3.76****
SP-PE	7.18 ± 0.31****	83.53 ± 0.45****	3.21 ± 2.79****

Data were expressed in mean ± SEM (n = 8). One-way ANOVA, test was applied, followed by the Bonferroni test for statistical analysis.

^#^ ^###^ *p* < 0.0001; compared with control group, ***p < 0.001, ****p < 0.0001; compared with CCl4 group.

### 3.4 SP extracts effect on inflammatory cytokines

In this study, the mRNA levels of the inflammatory cytokines IL-1β, IL-6, TNF-α, and fibrotic markers, α-SMA, TGF-β1, Smad2, Smad3 and COL1A1 were measured in the rats’ liver tissues. In rats’ liver tissue, the CCl4 group showed a significant elevation (*p* < 0.0001) in the mRNA expression levels of IL-1β, IL-6, TNF-α, α-SMA, TGF-β1, Smad2, Smad3 and COL1A1 with values of 3.32-, 4.86-, 5.63-, 7.82-, 8.97-, 6.05-, 5.42- and 15.1-fold, respectively. Whereas, silymarin, SP-AQ, SP-E, and SP-PE groups showed downregulation of IL-1β mRNA levels: 0.92-fold, 2.70-fold, 1.94-fold, and 1.47-fold, respectively. The downregulation of IL-6 was 1.32-, 3.57-, 2.49-, and 2.17-fold, respectively; TNF-α was 1.50-, 3.85-, 2.86-, and 2.33-fold, respectively; α-SMA was 1.15-, 4.69-, 1.48-, 1.20-fold respectively; TGF-β1 was 1.25-, 4.52-, 2.73-, and 2.10-fold, respectively; of Smad2 was 1.55-, 3.55-, 2.13- and 1.46-fold respectively; of Smad3 was 5.42-, 1.65-, 3.47-, 2.36-fold and 1.93-fold respectively and of COL1A1 was, 2.44-, 9.62, 5.28 and 3.87 -fold respectively, in the liver tissue (Figure 3). A significant decrease in mRNA expression of inflammatory, IL-1β, IL-6, TNF-α, and fibrotic markers α-SMA, TGF-β1, Smad2, Smad3 and COL1A1 was observed in both rats’ liver tissue of SP treated groups compared to the CCl4 group. When comparing various SP extracts, the SP-PE extract showed the greatest decrease (*p* < 0.0001) in mRNA expression levels of inflammatory and profibrotic genes, comparable to the silymarin group (*p* < 0.0001), followed by the SP-E extract (*p* < 0.001) and the SP-AQ extract (*p* < 0.01).

**FIGURE 3:**
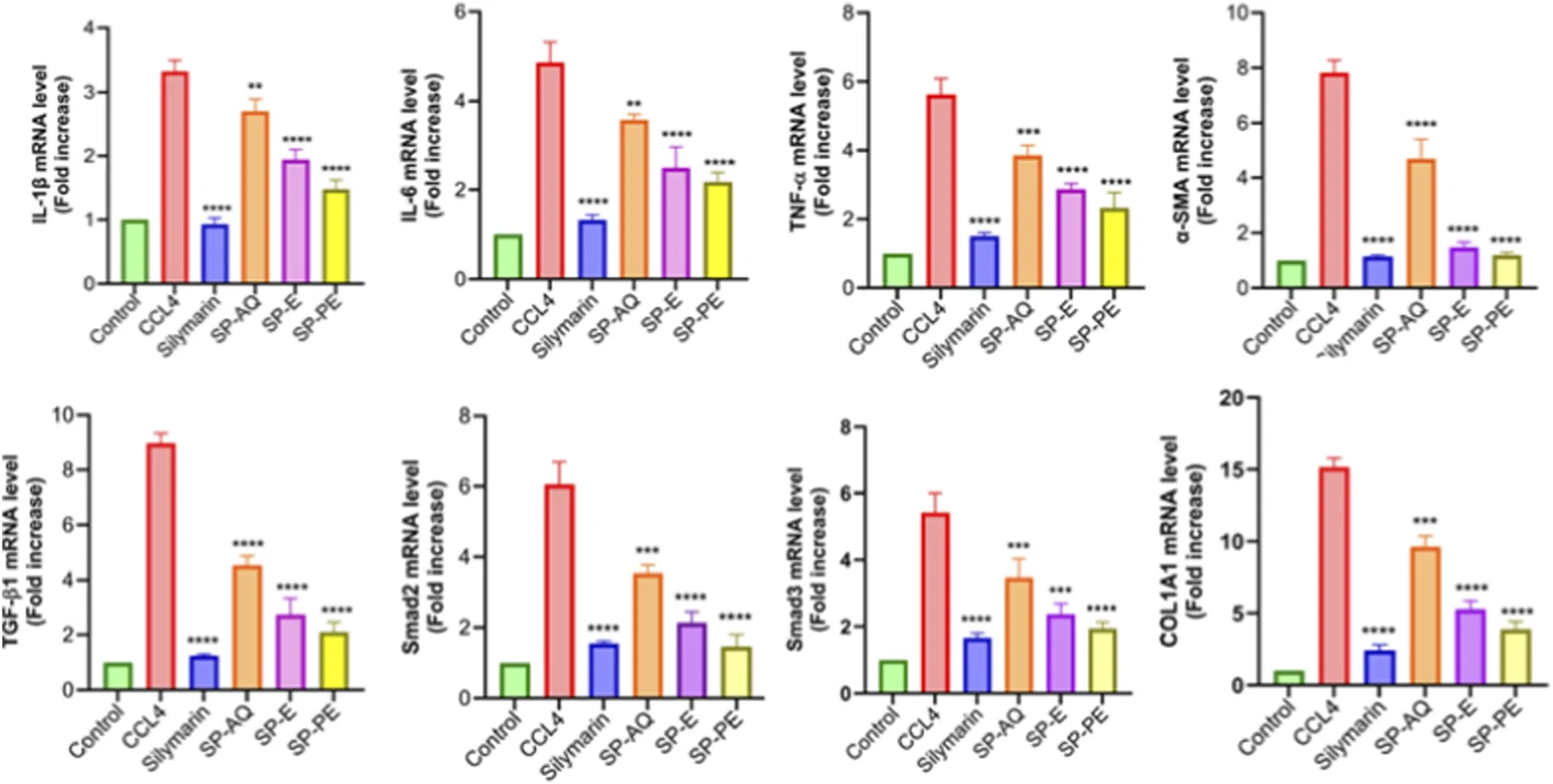
The effect of SP on the mRNA expression levels of inflammatory genes (IL-1β, IL-6, TNF-α) and profibrotic genes (TGF-β1, Smad2, Smad3, α-SMA, COL1A1) was measured in rat liver tissue (n = 8) of experimental groups that were analyzed in technical triplicate (n = 3) during the RT-qPCR assay. **p < 0.01, ***p < 0.001, ****p < 0.0001; compared to CCl4 group.

### 3.5 Histological examination

Hepatic histopathological observation of rats’ liver tissues by H&E stain was performed to observe the diseased fibrotic area of liver tissue and liver architecture. The fibrotic specialized stain markers, Van-Gieson’s stain, was used to stain type I collagen, and Masson’s trichome stain was used to stain total collagen by prominently staining type I collagen and type III collagen. The macroscopic examination of the liver in the CCl4 group revealed discoloration and white spots on its surface. Among the SP extracts treated groups, SP-AQ group showed an increased number of white spots, the SP-E group showed a lower number, and the SP-PE group revealed a marked reduction in white spots. The silymarin group showed a small white spot on the liver tissue (Figure 4A). The macroscopic examination of livers revealed that the SP-PE extract exhibited the highest hepatoprotective effect, followed by SP-E extract and SP-AQ extract.

**FIGURE 4:**
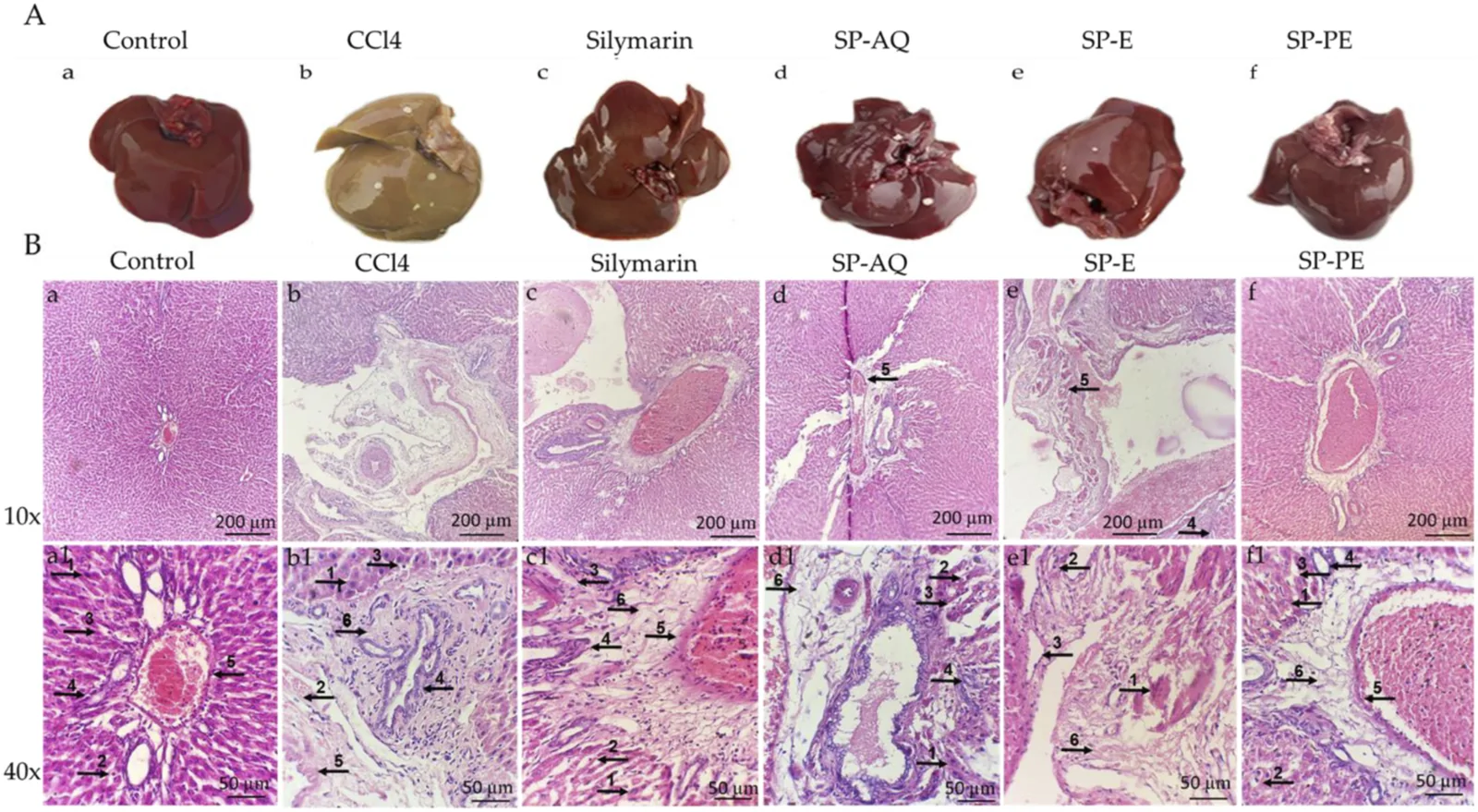
Effect of SP extracts on histopathological changes in the rat’s liver tissue with CCl4-induced liver fibrosis. **(A)** Gross liver morphology in different rat experimental groups; **(a)** The control group showed a normal liver, **(b)** CCl4 group showed prominent white spots across liver with discoloration, **(c)** Silymarin group showed minimal white spots, **(d)** SP-AQ group showed prominent white spots, **(e)** SP-E group showed a few white spots, **(f)** SP-PE group showed a small white spot. **(B)** H&E stained liver architecture in (a-a1) control group showed normal liver architecture, (b-b1) CCl4 group showed severe liver damage, (c-c1) silymarin group showed minimal liver damage, (d-d1) SP-AQ group showed moderate liver damage, (e-e1) SP-E group showed less moderate liver damage, (f-f1) SP-PE group showed mild liver damage. The arrow with labelled number indicates following 1, hepatocyte; 2. Kupffer cells; 3. Sinusoids; 4. Bile duct; 5. Portal vein; 6. fibrosis. The magnification scale bars are 50 μm at 10X and 200 μm at 40X.

#### 3.5.1 H&E staining of liver tissues

The rat liver section of the control group showed linear parenchyma with hepatocytes arranged in a single layer, trabecular, and limiting plates were intact. Fatty changes were not prominent, sinusoids were not congested and KCs were observed. Sinusoids were not congested. Intralobular and portal fibrosis were not observed (Figures 4A,B–a1). The CCl4 group showed enlargement of portal tracts with the severity of portal fibrosis. Focal activity was observed. A moderate level of chronic portal inflammation and dilation of bile ducts were revealed. The portal vein demonstrated thickened walls. Hepatocytes with feathery degenerations, prominent KCs and mildly congested sinusoids were shown (Figure 4B, b-b1). The silymarin group liver section showed portal tracts with mild portal fibrosis and mild chronic portal inflammation. Portal veins were observed to be thick-walled and congested. Hepatocytes showed no architectural distortion and fatty change was not observed. No prominent KCs and congested sinusoids were seen (Figure 4B, c-c1). The liver section of SP-AQ group demonstrated enlargement of portal tracts with a moderate to severe degree of liver fibrosis. Focal activity was also observed. Dilated portal veins and hepatocytes showing feathery degeneration were observed. Hyperplasia of KCs and unremarkable sinusoids were seen (Figure 4B, d-d1). The liver section of the SP-E group showed hepatic parenchyma with an arrangement of hepatocytes in regular single cell trabeculae. Focal inflammation with disorganized architecture was observed. Portal areas showed expansion due to some moderate fibrosis. A less moderate degree of chronic inflammatory infiltration was also observed (Figures 4B,E–e1). Mild dilatation of the portal vein and bile duct was seen. SP-PE group showed unremarkable linear liver architecture, with hepatocytes arranged in regular trabeculae. Intactness of the limiting plates was seen. A bit dilated bile duct was shown. A mild portal fibrosis was revealed. KCs and sinusoids were not prominent (Figure 4B, f-f1). SP extracts, notably SP-PE, attenuated CCl4-induced liver damage, inflammation, and necrosis, and restored the normal liver architecture.

#### 3.5.2 Fibrotic specialized staining of liver tissues

The rat’s liver tissues were stained with fibrotic specialized stains, Masson’s trichome stain that highlighted collagen (particularly type I and type III collagen) deposition by showing a blue color stain, and Van Gieson’s stain that highlighted collagen fiber (mainly type I collagen) by a reddish pink color stain. The control group liver appeared normal with no collagen deposition. In the CCl4 group, portal to portal and portal to central fibrosis were seen, as confirmed by Masson’s trichome stain and Van Gieson’s stain. In the silymarin group, the portal tract showed minimal portal fibrosis, and no portal to portal or portal to central fibrosis was seen. In SP-AQ group, a moderate to severe degree of portal and periportal fibrosis was seen. In SP-E group, portal areas were expanded by moderate fibrosis extending into lobules and less moderate fibrosis was seen. In SP-PE group, mild portal fibrosis was observed, and no portal to portal or portal to central fibrosis was seen (Figures 5A,B). At all stages of liver fibrosis, the extracellular matrix is predominantly composed of collagen types I and III. The activated HSCs and myofibroblasts cause overproduction and deposition of these collagen types I and III in central veins, fibrous septa, and portal areas (Mak et al., 2012). In histopathological findings, the SP extracts showed recovery from CCl4-induced liver fibrosis in rats, with SP-PE extract showing the most efficient recovery, comparable to the standard drug silymarin, SP-E extract moderate, and SP-AQ extract the least.

**FIGURE 5:**
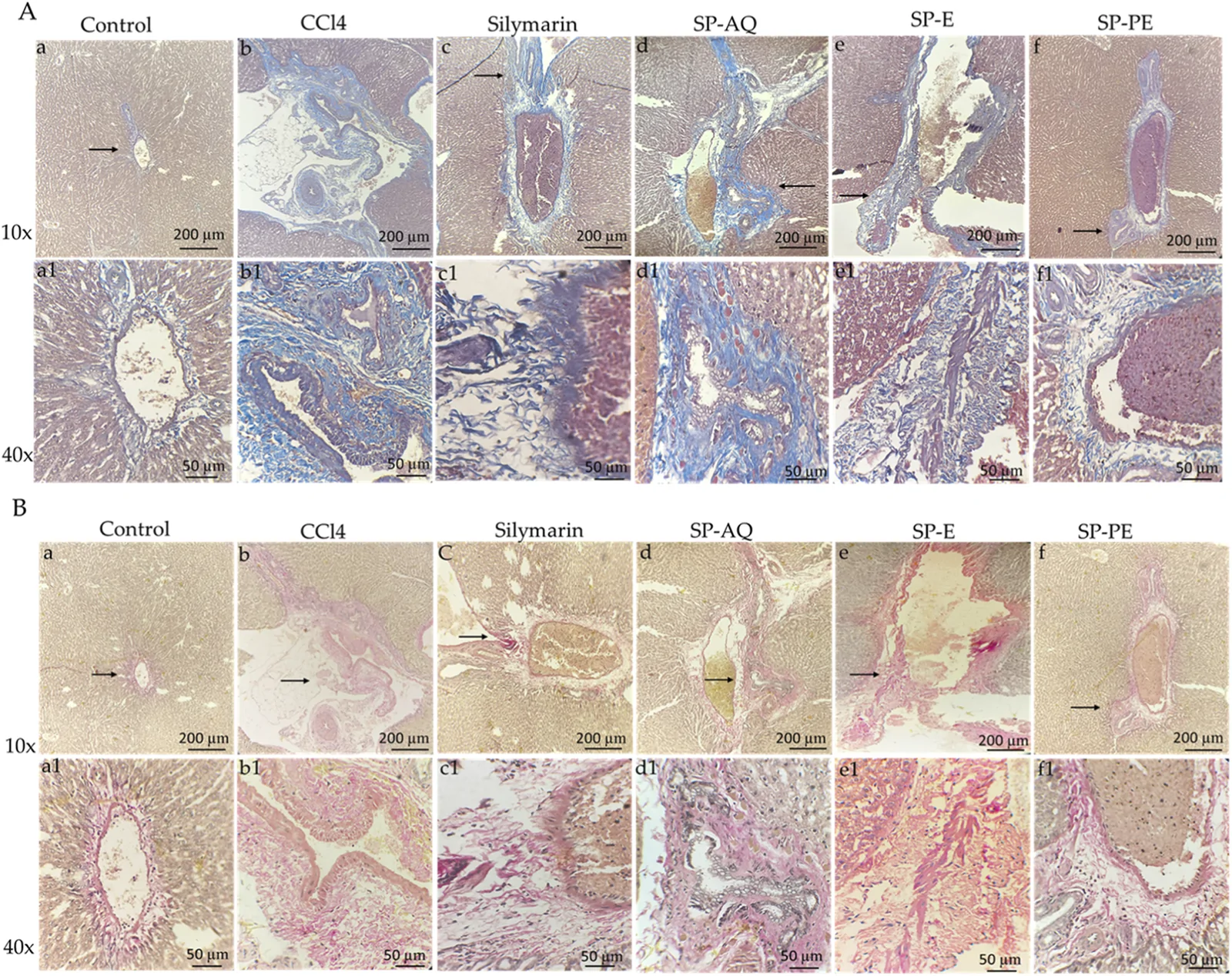
The effect of SP extracts on collagen accumulation in rats’ liver tissue were assessed by staining with fibrotic specialized stains, **(A)** Masson’s trichrome stain (blue color), that showed total collagen (particularly type I and type III collagen) in fibrotic area and **(B)** Van Giesson’s stain (reddish pink color) that showed type I collagen in (a-a1) control group with no fibrosis, (b-b1) CCl4 group showing severe fibrosis, (c-c1) silymarin group showing least fibrosis, (d-d1) SP-AQ group showing moderate to severe fibrosis, (e-e1) SP-E group showing moderate fibrosis and (f-f1) SP-PE group showing mild fibrosis. The magnification scale bars are 50 μm at 10X and 200 μm at 40X.

### 3.6 GC-MS phytochemical profile

The phytoanalysis of various SP extracts, namely, SP-AQ, SP-E, and SP-PE, using GC–MS revealed a wide diversity of bioactive phytoconstituents. A total of 55 compounds were identified across all three extracts, and their retention order along with percentage composition is presented in Table 5.

**TABLE 5:** Phytochemical constituents and their relative contents from various SP extracts.

No.	Compounds	M.F.	RT	LRI_Exp_	LRI_Lit_	SP-AQ	SP-E	SP-PE
1	2,2-Dimethyl-1,3-dioxolane-4-methanol	C_6_ H_12_ O_3_	2.47	956	947	0.5	-	1.0
2	**2-Furaldehyde, O-methyloxime**	**C**_**6**_ **H**_**7**_ **N O**_**2**_	**2.5**	**958**	**-**	**1.8**	**0.9**	**7.8**
3	5-Methyl-2-furancarboxaldehyde	​	2.63	978	964	0.7	0.71	-
4	Phenol	C_6_ H_6_ O	2.66	984	981	2.4	1.5	-
5	**2,4-Dihydroxy-2,5-dimethyl-3(2H)-furan-3-one**	**C**_**6**_ **H**_**8**_ **O**_**4**_	**2.72**	**992**	**989**	**3.7**	**1.7**	**-**
6	Benzyl chloride	C_7_ H_7_ Cl	2.93	1029	1023	1.7	1.2	0.7
7	**Benzyl alcohol**	**C**_**7**_ **H**_**8**_ **O**	**3.04**	**1046**	**1036**	**4.1**	**4.0**	**0.2**
8	1-(3-Methoxypropyl)pyrrolidine	C_8_ H_17_ N O	3.22	1079	1072	0.9	-	-
9	Furan-2,5-dicarbaldehyde	C_6_ H_4_ O_3_	3.27	1083	1076	0.7	-	-
10	Benzenemethanethiol	C_7_ H_8_ S	3.39	1100	1092	0.5	1.3	-
11	***Syn*-benzaldoxime methyl ether**	**C**_**8**_ **H**_**9**_ **N O**	**3.45**	**1112**	**-**	**1.0**	**2.5**	**3.8**
12	Maltol	C_6_ H_6_ O_3_	3.49	1122	1110	1.8	1.2	-
13	Phenylethyl alcohol	C_8_ H_10_ O	3.59	1126	1116	1.7	1.8	2.9
14	Benzyl isocyanate	C_8_ H_7_ N O	3.66	1131	1131	2.3	1.8	-
15	**Phenylacetonitrile**	**C**_**8**_ **H**_**7**_ **N**	**3.73**	**1148**	**1144**	**4.3**	**2.7**	**-**
16	2,3-Dihydro-3,5-dihydroxy-6-methyl-4H-pyran-4-One	C_6_ H_8_ O_4_	3.77	1160	1150	-	0.7	-
17	Benzoic acid	C_7_ H_6_ O_2_	3.82	1168	1172	1.4	0.6	0.5
18	**2,3-Dihydrobenzofuran**	**C**_**8**_ **H**_**8**_ **O**	**4.16**	**1223**	**1224**	**5.6**	**5.0**	**-**
19	5-Hydroxymethylfurfural	C_6_ H_6_ O_3_	4.23	1230	1232	1.0	0.5	-
20	2-Coumaranone	C_8_ H_6_ O_2_	4.37	1246	1249	2.3	1.3	-
21	**Phenylacetic acid**	**C**_**8**_ **H**_**8**_ **O**_**2**_	**4.43**	**1250**	**1262**	**0.6**	**-**	**9.2**
22	Phellandral	C_10_H_16_O	4.57	1282	1281	0.5	-	-
23	**4-Methoxybenzyl alcohol**	**C**_**8**_ **H**_**10**_ **O**_**2**_	**4.71**	**1290**	**1293**	**4.2**	**4.9**	**-**
24	**Proline, N-methyl-, butyl ester**	**C**_**10**_ **H**_**19**_ ** N O**_**2**_	**4.75**	**1302**	**1304**	**3.7**	**4.6**	**-**
25	**Benzyl thiocyanate**	**C**_**8**_ **H**_**7**_ **N S**	**5.25**	**1383**	**1339**	**26.7**	**32.9**	**3.3**
26	2-Phenylacetamide	C_8_ H_9_ N O	5.35	1410	1402	0.7	-	-
27	N-Benzylformamide	C_8_ H_9_ N O	5.44	1411	1402	1.8	1.2	-
28	2-Mercapto-nicotinic acid methyl ester	C_7_ H_7_ N O_2_ S	5.66	1438	1436	0.8	-	-
29	2-(3-Hydroxyphenyl)acetonitrile	C_8_ H_7_ N O	5.9	1495	1494	0.7	1.1	-
30	Cyclohexanecarboxylic acid, 2-methylpentyl ester	C_13_ H_24_ O_2_	6.17	1526	1524	1.8	-	-
31	Mandelamide	C_8_ H_9_ N O_2_	6.25	1538	1536	-	1.3	-
32	2,6-Dimethoxy-4-vinylphenol	C_10_ H_12_ O_3_	6.46	1568	1567	-	0.5	-
33	*m*-Methoxybenzylisothiocyanate	C_9_ H_9_ N O S	6.72	1620	1619	0.9	-	-
34	4-Methoxybenzyl isothiocyanate	C_9_ H_9_ N O S	6.81	1632	1636	-	0.5	-
35	4-Hydroxy-3,5-dimethoxybenzonitrile	C_9_ H_9_ N O_3_	7.14	1688	1673	2.3	0.6	-
36	**4-Propenyl-2,6-dimethoxyphenol**	**C**_**11**_ **H**_**14**_ **O**_**3**_	**7.26**	**1706**	**1704**	**2.0**	**3.0**	**24.1**
37	2-(Ethylsulfonyl)methyl)-phenol	C_9_ H_12_ O_3_ S	7.29	1711	-	2.1	1.6	-
38	N-Benzylidenebenzylamine	C_14_ H_13_ N	7.60	1770	1761	0.5	-	-
39	4-(2-Hydroxyethyl)-2,6-dimethoxyphenol	C_10_ H_14_ O_4_	7.62	1784	1786	0.5	-	-
40	Syringic acid	C_9_ H_10_ O_5_	7.68	1819	1821	0.6	0.5	-
41	Diethylone	C_14_ H_19_ N O_3_	7.87	1827	1824	-	0.9	-
42	Diisobutyl phthalate	C_16_ H_22_ O_4_	8.18	1882	1869	-	-	0.7
43	Dibutyl phthalate	C_16_ H_22_ O_4_	8.58	1962	1965	-	0.6	-
44	Macaridine	C_13_ H_13_ N O_2_	8.67	1977	1971	1.1	-	-
45	**N-(3-methylphenyl)thiophene-2-carboxamide**	**C**_**12**_ **H**_**11**_ ** N O S**	**8.98**	**2039**	**2037**	**-**	**-**	**5.9**
46	Elaidic acid	C_18_ H_34_ O_2_	9.45	2139	2144	-	1.2	-
47	**4-Methyl-1-(4-methylphenyl)-5-phenyl-1 H-imidazole**	**C**_**17**_ **H**_**16**_ **N**_**2**_	**9.78**	**2225**	**2226**	**-**	**-**	**7.2**
48	8,11,14-Eicosatrienoic acid, methyl ester	C_21_ H_36_ O_2_	9.88	2236	2239	-	-	1.0
49	Glycidyl palmitate	C_19_ H_36_ O_3_	10.12	2291	2294	1.0	0.6	-
50	Phenylalanylleucine	C_15_ H_22_ N_2_ O_3_	10.36	2345	2349	-	-	0.5
51	Bis(2-ethylhexyl) phthalate	C_24_ H_38_ O_4_	11.28	2572	2535	-	-	0.8
52	1,2-bis(4-hydroxy-3-methoxyphenyl)ethylene	C_16_ H_16_ O_4_	11.44	2616	2654	-	-	1.4
53	Terephthalic acid, 6-methylhept-2-yl octyl ester	C_24_ H_38_ O_4_	12.03	2753	2756	-	-	0.5
54	**(Z)-13-Docosenamide**	**C**_**22**_ **H**_**43**_ ** N O**	**12.14**	**2791**	**2793**	**-**	**-**	**4.0**
55	**(3*β*,24*S*)-stigmast-5-en-3-ol**	**C**_**29**_ **H**_**50**_ **O**	**15.64**	**3371**	**3368**	**-**	**-**	**3.9**

Compounds more than 3.0 % are highlighted with bold.

Additionally, the GC chromatograms of each extract, together with the identified compounds, are shown in Figure 6.

**FIGURE 6:**
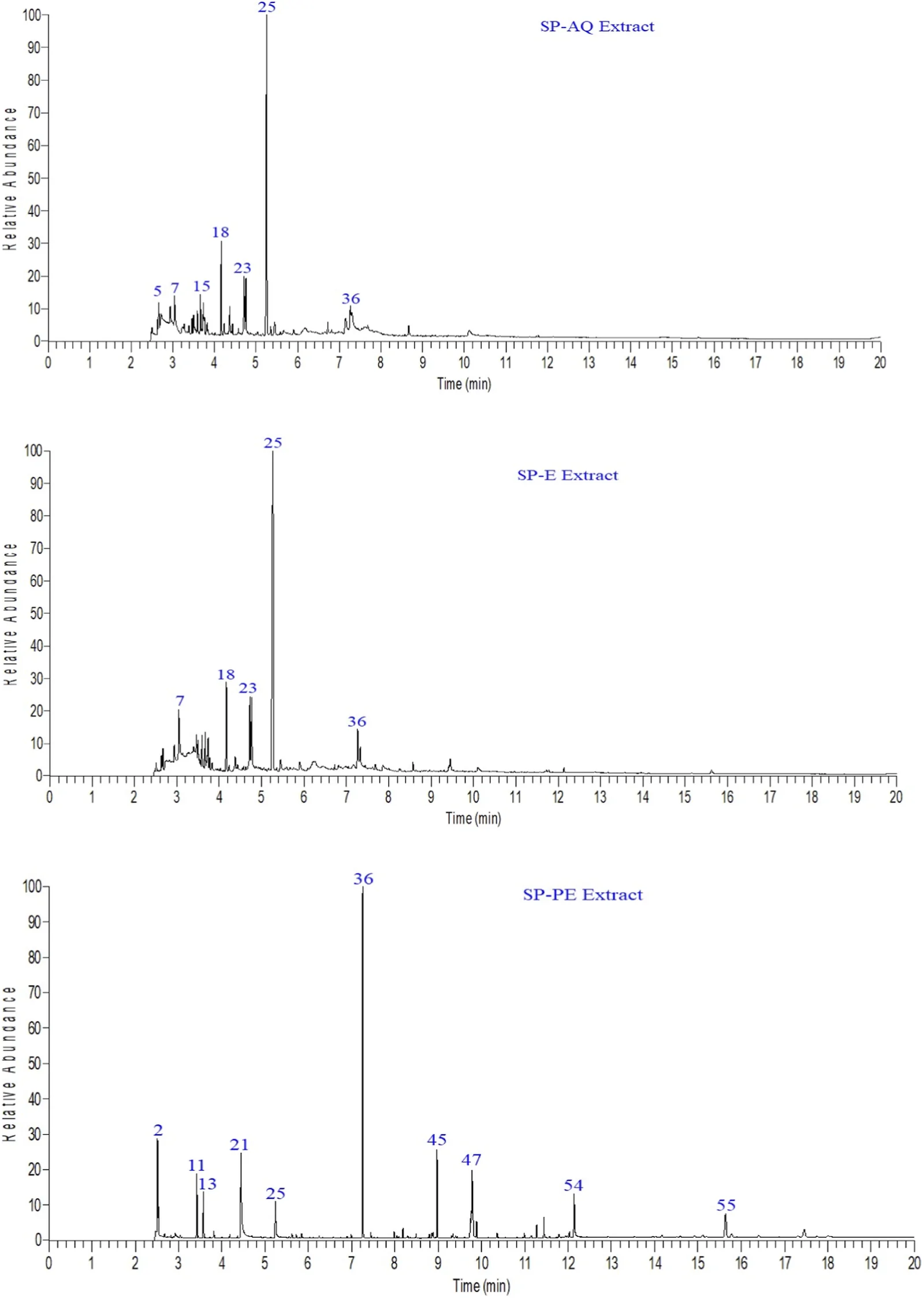
Total ion chromatogram (TIC) of SP various extracts by GC-MS analysis.

Among the 55 identified phytoconstituents, 38 compounds were detected in the SP-AQ extract, 33 compounds in the SP-E extract, and 20 compounds in the SP-PE extract. Detailed analysis showed that 8 phytoconstituents were common to all three extracts, whereas 28 compounds were unique or differentially distributed among the extracts. Of these, 11 compounds were specific to the SP-AQ extract, 7 were exclusive to the SP-E extract, and 10 were uniquely present in the SP-PE extract. Notably, the SP-AQ and SP-E extracts exhibited considerable similarity in their volatile phytochemical composition, whereas the SP-PE extract displayed a distinctly different profile in comparison to both SP-AQ and SP-E extracts. Specifically, 26 compounds were found to be common between SP-AQ and SP-E extracts, with the predominant constituents being benzyl thiocyanate (26.73% and 32.89%, respectively), 2,3-dihydrobenzofuran (5.64% and 5.03%, respectively), and 4-methoxybenzyl alcohol (4.17% and 4.92%, respectively). In contrast, only 10 compounds were shared between SP-AQ and SP-PE extracts, while merely 8 constituents were common between SP-E and SP-PE extracts. The SP-PE extract was characterized by the predominance of 4-propenyl-2,6-dimethoxyphenol (24.07%), phenylacetic acid (9.24%), 2-furaldehyde, O-methyloxime (7.77%), 4-methyl-1-(4-methylphenyl)-5-phenyl-1H-imidazole (7.15%), N-(3-methylphenyl)thiophene-2-carboxamide (5.89%), and (Z)-13-docosenamide (4.03%) (Figure 7).

**FIGURE 7:**
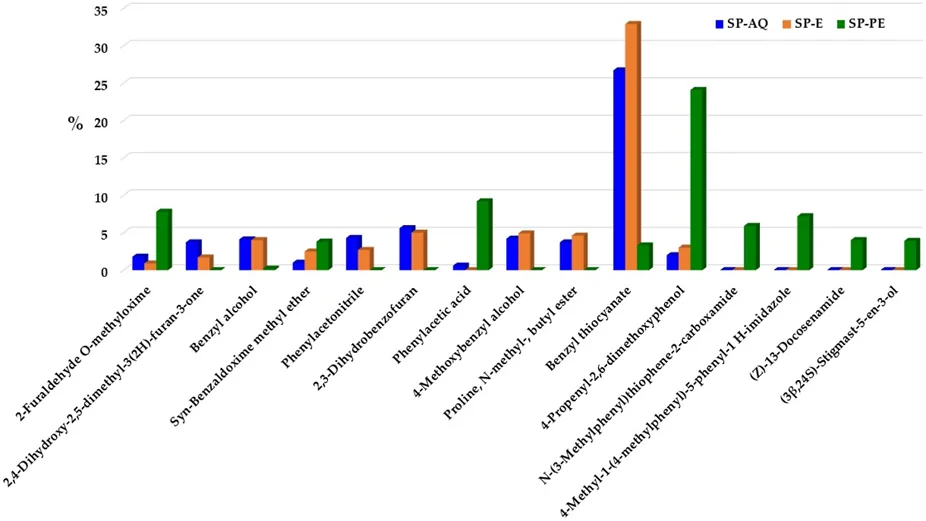
Major Phytoconstituents identified in various SP extracts identified in GC-MS analysis.

To improve the transparency and clarity of the data presentation, we have included higher-resolution chromatograms in the Supplementary Figures 1–3. In these chromatograms, nearly all compounds listed in Table 5 have been numbered and annotated. However, a few minor constituents could not be individually labeled because their signals originate from overlapping or unresolved peaks in the highly complex TICs, making their visual assignment impractical despite their successful identification through the deconvolution and spectral matching procedures.

### 3.7 Multivariate PCA analysis and PLS-DA analysis

Multivariate analysis of the SP-AQ, SP-E, and SP-PE extracts, based on relative peak area percentages of phytocompounds, revealed distinct separation among them. Principal Component Analysis (PCA) showed strong lustering pattern revealing the substantial differences in phytochemicals of SP extracts, explaining 90.83% of total variance (PC1 = 72.50%, PC2 = 8.33%) which shows that SP extracts yielded considerable uncommon phytocompounds, while PLS-DA demonstrated well-defined clustering (Component 1 = 65.2%, Component 2 = 10%). The SP-PE extract formed a distinct, isolated cluster, indicating a unique metabolite composition compared with the other extracts. Permutation testing finding suggested that the PLS-DA model was not overfit (R^2^ = 0.7197, Q^2^ = −0.4443), but the negative Q value indicated poor predictive ability (Figure 8).

**FIGURE 8:**
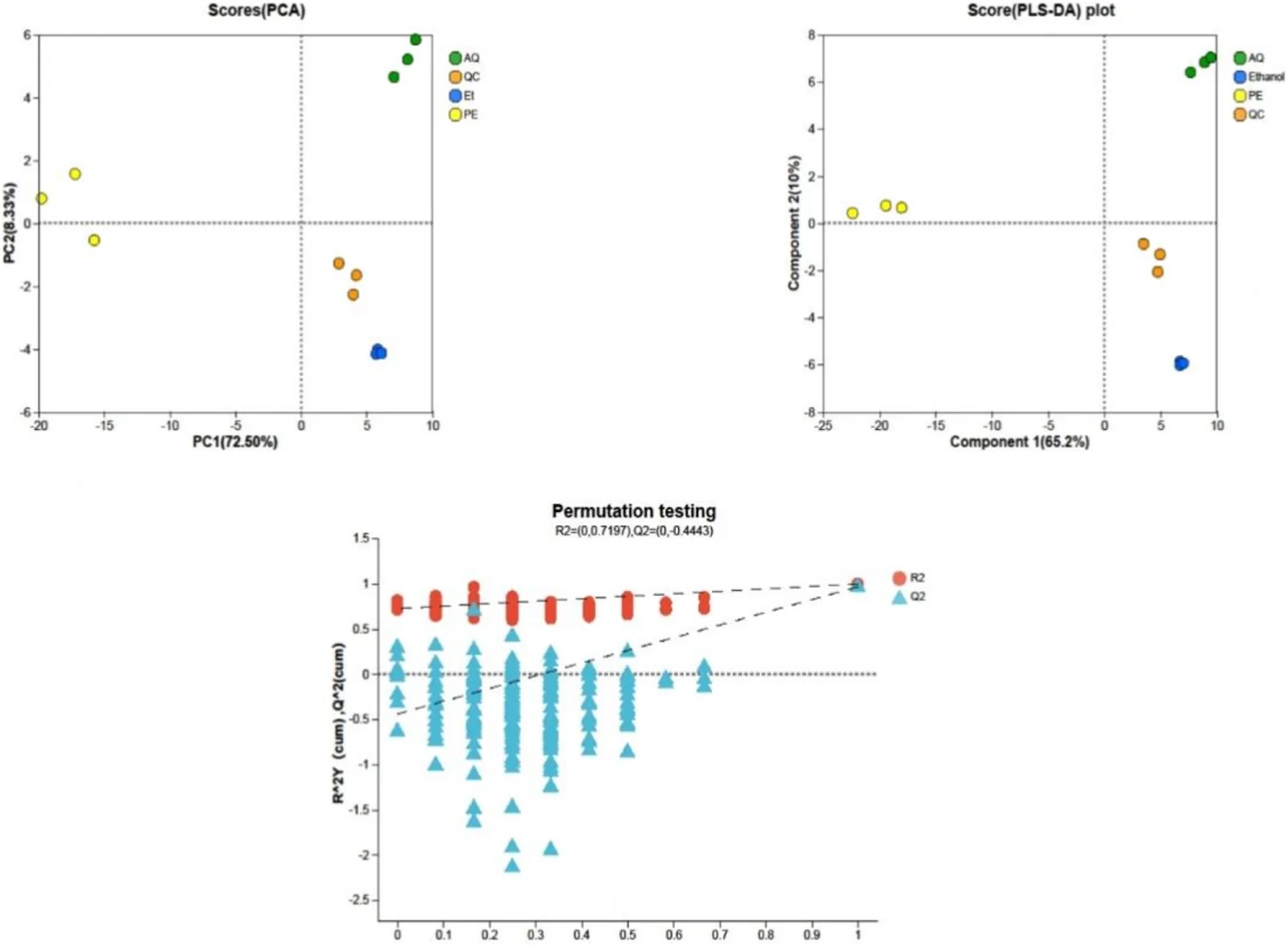
Multivariate PCA, PLS-DA analysis and permutation testing of phytochemicals of SP extracts based on GC-MS data.

## 4 Discussion

Natural plants have been reported to reduce oxidative stress and inflammation and are considered a therapeutically effective approach in treating liver diseases, including fibrosis (Niu et al., 2025). In the current study, we evaluated the *in vivo* comparative effects of SP extracts on chronic liver injury fibrosis in rats induced by CCl4, along with their phytochemical characterization. This study, determined the effects of SP extracts on rats’ body weight, liver weight, fibrotic markers and collagen deposition by fibrotic-specific staining. In a previous SP crude extract was evaluated for acute toxicity in mice and was found safe at 5000 mg/kg (Gebrehiwot et al., 2019). Based on previous *in vivo* acute hepatotoxicity studies that employed 100-500 mg/kg doses (Qureshi et al., 2016; Alaraj et al., 2021; Bokhary et al., 2022), we selected 400 mg/kg dose for SP extracts to achieve optimal effect in liver fibrosis. CCl4 is a well-known hepatotoxin that induces liver fibrosis in rats, making it a useful model for hepatoprotective studies that induce oxidative stress and inflammation, stimulate HSCs and promote the accumulation of excessive ECM and the release of profibrogenic mediators to develop liver fibrosis (Bai et al., 2024; Das et al., 2026a; Das et al., 2026b). The hepatotoxic, CCl4, is activated by liver microsomal enzyme to produce a free radical, trichloromethyl, CCl3-that reacts with oxygen and forms an intense reactive radical, trichloromethyl peroxyl (CCl300-), which causes lipid peroxidation by interacting with polyunsaturated fatty acids, resulting in severe liver injury, stimulation of inflammation and fibroblasts (Ullah et al., 2020). Chronic inflammatory conditions in rats cause weight loss by reducing food intake due to liver injury. Inflammatory conditions cause the liver to appear boggy, flabby, and heavier than a normal, healthy liver (Unsal et al., 2021). In the current study, in the CCl4 group, the decrease in rat body weight and the increase in liver weight and liver index were observed, which can be attributed to increased inflammation resulting from CCl4-induced hepatotoxicity. In contrast, SP extracts improved body weight in treated rats and showed an inconsiderable increase in liver weight and liver index, and demonstrated hepatoprotective activity, with less pronounced in the SP-AQ extract, modest in the SP-E extract, and most notably in the SP-PE extract, which may be attributed to reduction in the oxidative stress and inflammation related markers as evidenced by SP extracts findings of antioxidants and anti-inflammatory effects in the current study. The observed variation in hepatoprotective activity of SP extracts may be associated with differences in solvent polarity, as SP-PE was extracted in the non-polar solvent petroleum ether, SP-E was extracted in the intermediate polar solvent ethanol, and SP-AQ was extracted in the polar solvent distilled water, which affect the distinct phytochemical profiles of extracts, revealed by GC-MS analysis. The observed results are consistent with a previously reported study in which the medicinal plant *Lycium barbarum* showed hepatoprotective activity, improved body weight in rats, and an insignificant increase in liver weight and liver index in CCl4-induced liver fibrosis in rats (Gan et al., 2018). CCl4, a strong free radical, trichloromethyl (CCl3-), covalently binds with hepatocyte cell membrane proteins and lipids and destroys the cell integrity and its structure. This damage leads to the release of intracellular liver enzymes, such as ALT, AST, and ALP (Agrawal et al., 2016). Liver damage impairs hepatic protein synthesis, as assessed by globulin, albumin and Total protein and the liver excretory role is indicated by Total bilirubin (Jaishree and Badami, 2010). The present study showed that CCl4 causes a considerable elevation in liver biochemical parameters, AST, ALT, ALP, Total bilirubin, albumin, globulin and Total protein in rats serum, which is in line with a previous CCl4-induced liver fibrosis study in which CCl4 increased the serum liver biochemical parameters in rats (Aykaç et al., 2024). The decrease in elevated liver biochemical parameters indicates hepatoprotective activity (Aksoy et al., 2026). In the current study, the SP extracts significantly reduced the elevated levels of liver biochemical parameters and improved protein synthesis in the treated rat groups, with less significant effect in the SP-AQ extract group, modest activity in the SP-E extract group, and the most pronounced activity in the SP-PE extract group. This suggests the hepatoprotective activity of SP extracts, and these results are consistent with a previously reported study in which SP aqueous extract revealed hepatoprotective activity by reducing the elevated levels of ALT. AST, ALP and bilirubin in paracetamol-induced acute hepatotoxicity in rats (Alaraj et al., 2021). The covalent binding of the (CCl3-) radical leads to the deposition of lipid peroxidation end products, such as MDA, in cells, thereby disrupting the antioxidant defense system and reducing the activities of the antioxidative enzymes SOD and GPx, which increase in response to lipid peroxidation-mediated radical production in the liver. A lower-than-normal level of these antioxidant enzymes indicates the severity of liver injury (Wang et al., 2025). Excessive oxidative stress from free radical production causes liver toxicity and fibrosis (Ramos-Tovar and Muriel, 2020). SOD suppresses lipid peroxidation, and GPx degrades hydrogen peroxide (H_2_O_2_) and detoxifies lipid hydroperoxides, thereby protecting liver cells from oxidative stress-induced injury (Mooli et al., 2022). In the current study, CCl4-intoxicated rat group exhibited significant oxidative stress, as evidenced by increased MDA levels and decreased activities of the antioxidant enzymes SOD and GPx. In contrast, SP extract-treated rat groups showed the greatest decrease in MDA level and the most remarkable increases in SOD and GPx levels, which was most significant in the SP-PE group, considerable in the SP-E group, and least in the SP-AQ group. These results are in agreement with a previously reported study in which the SP aqueous extract showed increased levels of the antioxidant enzymes CAT and GPx and decreased MDA levels (Bokhary et al., 2022).The CCl4 intoxication induces KCs to secrete inflammatory cytokines, including TNF-α, IL-1β, and IL-6 (Muriel, 2009), which in turn activate HSCs to release TGF-β1, leading to increased ECM deposition and fibrosis in the liver by promoting the release of fibrotic mediators, α-SMA and COL1A1 (Hameed et al., 2022). The inflammatory cytokine IL-1β upregulates profibrogenic TGF-β1 and prolongs HSCs survival. TGF-β1 is a key profibrotic factor that induces the transdifferentiation of quiescent HSCs into myofibroblasts, thereby increasing ECM production and α-SMA expression and promoting liver fibrosis (Dawood et al., 2020). In HSCs, profibrotic factor TGF-β1 induces the collagen I and III transcription via the Smad-dependent signaling cascade. The Smad2 and Smad3 are downstream targets of TGF-β1 (Qu et al., 2015). TGF-β1 bind to the Smad2 and Smad3 receptors and activates Smad2 and Smad3 by phosphorylating them. TGF-β1/Smad signaling pathway activates the downstream cellular mechanism leading to HSCs cell survival and proliferation and induces many proinflammatory cytokines and other profibrotic mediators to promote liver fibrosis. The activated Smads enter the cell nucleus and regulate the transcription of TGF-β-dependent genes (Chhimwal et al., 2020). In the current study, the CCl4-intoxicated rat group showed significant upregulation of the mRNA expression levels of inflammatory markers (IL-1β, IL-6, TNF-α) and profibrogenic markers (α-SMA, TGF-β1, Smad2, Smad3, COL1A1 in liver tissues. In contrast, the SP extracts downregulated mRNA levels of inflammatory and profibrotic genes, which suggested SP extracts showed anti-inflammatory and antifibrotic effects by modulating gene expression associated with the TGF-β1/Smad signaling pathway. These findings suggest the hepatoprotective activity of SP extracts, with the most pronounced effect in SP-PE extract and the least by SP-AQ extract. The SP extracts impact on mRNA quantification of genes related to inflammation and fibrosis suggests that SP extracts have modulated the genes expression levels related with TGF-β1/Smad signaling, which can be attributed to their antioxidant effects as evidenced by reduction in oxidative stress markers and differentiated phytochemical profile of extracts due to differences in solvent selection for SP extracts. CCl4-induced liver fibrosis is characterized by increased oxidative stress, which activates inflammatory and profibrotic signaling pathways. SP extracts showed a parallel trend in modulating the inflammatory and profibrotic gene expression levels, which can be attributed to their shared underlying biological influence on these genes and the presence of common phytochemicals identified in the SP extracts although these extracts differ in solvent polarity**.** The current study anti-inflammatory results of SP extracts are in line with the previous SP study in which SP aqueous downregulated IL-6 and TNF-α mRNA levels in ethanol-induced gastric ulcer in rats (Lebda et al., 2018). CCl4 intoxication causes the disruption of liver appearance and impair the liver architecture. In the current study, the CCl4 group, rats’ liver showed discoloration and white spots across the liver surface, indicating liver degeneration with focal necrosis and fibrotic nodule formation, which reveals the subchronic to chronic stage of CCl4-induced liver injury (Krishna, 2017). In contrast, SP extracts treated rat groups were observed with normal color of liver and reduced white spots were visible across the liver surface, which was more prominent in the SP-PE extract group, minimal in SP-E extract group and considerable in SP-AQ extract group. The histopathological findings in rats using H&E staining in CCl4 group revealed severe disruption of liver architecture, with necrosis, severe inflammation, dilated bile ducts, congested sinusoids, prominent KCs and portal fibrosis, which are characteristic of chronic liver injury fibrosis (Mak et al., 2012). The SP extracts treated rat groups showed the normalization of liver architecture, most effectively with the SP-PE extract, to a moderate extent with the SP-E extract, and lesser with the SP-AQ extract. These results are in line with a previous study in which SP aqueous root extract restored liver architecture in paracetamol-induced hepatorenal damage (Alaraj et al., 2021), and with another study demonstrating SP aqueous root extract’s hepatoprotective activity in cyclophosphamide-induced hepatorenal damage (Bokhary et al., 2022). This suggests that SP extracts have demonstrated hepatoprotective activity in treated rats by restoring liver architecture. The fibrotic-specific Masson’s trichrome stain was used to stain total collagen, with greater prominence of type I collagen and type III Collagen, and Van Gieson’s stain was used to primarily stain type I Collagen. In CCl4 group was observed with portal to portal and portal to central fibrosis with extensive collagen accumulation. Among SP-extracts, SP-AQ showed moderate portal to periportal fibrosis with moderate collagen deposition and less moderate in the SP-E extract group and less in the SP-PE extract group, which reveals the hepatoprotective activity of SP extracts, most remarkably by SP-PE extract. These results on reducing collagen deposition are in line with a previous study in which Moringa oleifera extract showed antifibrotic effects by reducing total collagen deposition using Masson’s trichrome staining (Baky et al., 2025) and collagen I using Van Gieson’s staining (Zhang et al., 2022). This suggests that SP extracts demonstrated antifibrotic effects in rats most effectively with the SP-PE extract, moderately with the SP-E extract, and less with the SP-AQ extract. The hepatoprotective activity of SP extracts are also in line with another study in which Ginger extract demonstrated antifibrotic activity in induced liver fibrosis induced by CCl4 in olive oil 1:1 v/v of 8 weeks study (Almatroodi et al., 2020). Although the SP-PE extract showed mild histological alterations, it exhibited overall greater hepatoprotective effects, as evidenced by biochemical, molecular, and histological findings, approaching close to the standard drug, silymarin. The present study findings suggest that SP extracts, SP-AQ, SP-E, and SP-PE have exerted hepatoprotective effects in CCl4-induced liver fibrosis in rats, as evidenced by improvement in body weight with insignificant increase in liver index, attenuation in the liver biochemical parameters, oxidative stress markers, inflammatory and profibrotic genes expressions levels, and histological alterations and collagen deposition in damaged liver, which reveals SP extracts has exerted antifibrotic activity. The SP extracts showed consistent hepatoprotective effects with SP-PE > SP-E > SP-AQ, suggesting coordinated biological effects across all evaluated parameters rather than independent effects. In the present study, the observed hepatoprotective effects strongly indicate that extraction solvents of different polarities selectively enriched distinct bioactive phytochemicals, which in turn could affect the hepatoprotective and anti-fibrotic activity. The superior efficacy of SP-PE extract than the remaining SP extracts might be due to differences in bioactive compounds. Plants are a rich source of phytochemicals with proven therapeutic potential against liver fibrosis, owing to their antioxidant and anti-inflammatory properties (Xu et al., 2023). In the current study, the phytochemical profile of SP extract from the root part was investigated using GC-MS. Among the 55 identified phytoconstituents, 8 were common in all SP extracts, while 47 compounds were uncommon, of which 28 compounds were unique or differentially distributed among the extracts, which indicated the SP extracts separation. The phytoconstituent benzyl thiocyanate was found primarily in the SP-AQ and SP-E extracts at 26.73% and 32.89%, respectively, while SP-PE showed 3.3% of this compound. This result is also consistent with a previous study that reported the presence of benzylisothiocyanate in the SP root extract (Abdel-kader et al., 2017). Our study revealed the presence of benzylthiocyanate in SP extracts, a structural isomer of benzylisothiocyanate. The Phytochemical profile of SP-PE extract showed mainly the 4-propenyl-2,6-dimethoxyphenol (24.07%) along with other major phytoconstituents, phenylacetic acid (9.24%), 2-furaldehyde, O-methyloxime (7.77%), 4-methyl-1-(4-methylphenyl)-5-phenyl-1H-imidazole (7.15%). Various classes of phytoconstituents, including thiocyanates (Xu et al., 2009), phenolics (Houser et al., 2012; Liu et al., 2023), organic acids (Zheng et al., 2021), and imidazole derivatives (Alghamdi et al., 2021), isolated from different plant sources, have been reported to exhibit antioxidant, anti-inflammatory, and antifibrotic activities. In the present study, phytochemical analysis of different SP extracts revealed the presence of several similar phytoconstituents in SP-AQ, SP-E, and SP-PE extracts. The phthalate derivatives, dibutyl phthalate in SP-E extract and diisobutyl phthalate and bis(2-ethylhexyl) phthalate in the SP-PE extract were detected in trace amounts. These compounds are commonly reported as contaminants originating from plastic laboratory ware, solvent storage containers, septa, tubing, or GC-MS consumables. We do not attribute any biological activity of the extracts to these phthalate derivatives, nor do we consider them definitive phytoconstituents of *Salvadora persica* without further confirmation by targeted analytical methods. Correspondingly, the biological activity studies revealed that SP-AQ showed mild improvement, whereas SP-E and SP-PE extracts showed significant recovery, with the most pronounced recovery by SP-PE extract from CCl4-induced liver fibrosis in rats through antioxidant and anti-inflammatory activities, modulating the transcriptional expression levels of genes related to the TGF-β1/Smad signaling pathway. Comparative hepatoprotective study of SP extracts revealed the most pronounced antifibrotic activity by SP-PE extract, modest by SP-E extract and least by SP-AQ extract. The results showed that the SP-PE extract was consistently superior to the traditionally used SP-AQ extract. According to our data, this conventional preparation may not be the best in extracting the most pharmacologically active constituents. This shows the significant role of solvent selection in extraction and elucidates the differentiated phytochemicals in extracts obtained with different solvents. The GC-MS analysis of the SP-PE extract in a non-polar solvent identified significant amounts of phytochemicals that worked synergistically. The superior hepatoprotective and antifibrotic effects of SP-PE could be linked to its unique phytochemical composition, as identified by GC-MS. The present study did not examine the biological activity and mechanistic roles of individual components of SP-PE however, major phytochemicals, 4-propenyl-2,6-dimethoxyphenol (phenol), and other dominated phytochemicals, phenylacetic acid (aromatic carboxylic acid), 2-furaldehyde (heterocyclic aldehydes), O-methyloxime (oxime) and 4-methyl-1-(4-methylphenyl)-5-phenyl-1H-imidazole (imidazole derivative), were identified in SP-PE which have been reported for their antioxidant and anti-inflamamtory effects. Furthermore, it is noteworthy that 4-propenyl-2,6-dimethoxyphenol was identified as the major constituent of the SP-PE extract and was also detected in appreciable amounts in both SP-E and SP-AQ extracts. This compound may have contributed substantially to the hepatoprotective activity exhibited by the SP extracts. On the other hand, benzyl thiocyanate (thiocyanate), which has been reported to have antioxidant activity, was identified in higher relative amounts in the SP-AQ and SP-E extracts than in the SP-PE extract, but its biological activity was comparatively lower. The present results suggest that the observed hepatoprotection is not solely attributable to a single phytochemical. Instead, their effects are likely due to the combined, synergistic actions of several bioactive compounds identified in the extracts. Moreover, variations in the biological activities of the extracts could have been due to differences in phytochemical composition resulting from the extraction solvent. Hence, bioactivity guided fractionation and molecular mechanism-based studies of the isolated constituents are desirable to identify the main bioactive compounds and elucidate their molecular mechanisms of action. Therefore, the observed hepatoprotective effects may be attributed to the phytoconstituents identified in the various SP extracts. Further research is recommended using fractionation, and evaluation of isolated compounds to separate the individual and synergistic actions of phytoconstituents. The major compound needs to be evaluated to quantify protein markers associated with the TGF-β1/Smad pathway. The multivariate PCA analysis confirmed distinct clustering in the SP-PE extract, and the PLS-DA analysis also confirmed separation among SP extracts. Permetuation testing validated the results of PLS-DA multivariate analysis. The score plot of the PLS-DA was primarily used to visualize differences among the SP-PE, SP-E, and SP-AQ extracts. The SP-PE extract clustered in a distinct area, indicating a different metabolite profile; however, the negative Q^2^ value indicated that the model was not very predictive. This suggested that PLS-DA served as exploratory, indicating extract separation instead of a predictive model. This separation was considered a preliminary indication of compositional differences rather than a definitive classification. These results are in line with the results obtained by the GC-MS analysis, which showed a unique phytochemical profile in the SP-PE extract, which might be potential candidates for additional preclinical, mechanistic, and clinical studies to elucidate their therapeutic efficacy as hepatoprotective agents. However, further investigation is necessary to evaluate the hepatoprotective efficacy of the pure compound isolated from the SP-PE extract.

## 5 Conclusion

The present study showed that various SP extracts displayed significant hepatoprotective activity against CCl4-induced liver fibrosis in the rat model. The results revealed that SP extracts protect liver tissue from the hepatotoxic effects of CCl4 by improving body weight without noticeably increasing liver weight and liver index. SP reduced hepatotoxicity by lowering elevated serum liver biochemical markers and lipid peroxidation, and by enhancing the activity of antioxidant enzymes. SP extracts decreased the mRNA levels of inflammatory and profibrotic genes in rat liver tissue associated with the TGF-β1/Smad signaling pathway. SP extracts histopathologically restored the damaged architecture of liver tissue by alleviating collagen deposition. Moreover, phytochemical analysis of the SP extracts revealed marked differences among SP-AQ, SP-E, and SP-PE. The polarity-dependent order of biological activity, SP-PE > SP-E > SP-AQ suggests that the SP-PE extract is enriched with non-polar bioactive phytochemicals, and the major phytochemicals recognized in GC-MS analysis may have exerted the synergistic effect, which might be attributed to the greater hepatoprotective and anti-fibrotic effects in rats. The multivariate analysis confirmed separation among SP extracts, with distinct clustering observed in the SP-PE extract. Notably, both biological and phytochemical investigations demonstrated the significant hepatoprotective potential of SP extracts against liver fibrosis through their antioxidant and anti-inflammatory activities, which reduce the expression of inflammatory and fibrotic genes related to the TGF-β1/Smad signaling pathway. Our study assessed transcript levels, and thus these results suggest transcriptional regulation, and not direct inhibition of pathway activity. The SP-PE extract exhibited the highest antifibrotic activity, comparable to the standard drug Silymarin. Therefore, the SP-PE extract may be considered a promising candidate for developing hepatoprotective agents to treat liver fibrosis. Further detailed studies focusing on the isolation and characterization of individual bioactive hepatoprotective compounds from the SP-PE extract are warranted to elucidate their roles in fibrosis and cirrhosis, as well as the underlying molecular signaling mechanisms.

### 5.1 Limitations of the study

The current study determined the SP extracts impact on gene expression related to the TGF-β1/Smad pathway; however, it could not provide additional experimental data, including quantification of proteins and Smad phosphorylation, to provide mechanistic evidence of SP extracts targeting the TGF-β1/Smad signaling pathway to elucidate the SP extracts post-translational role for clinical therapeutic efficacy. The interpretation of the histology could be strengthened by including the quantitative fibrosis scores in the study.

## Data Availability

The original contributions presented in the study are included in the article/Supplementary Material, further inquiries can be directed to the corresponding author.
