# Supplementary material for: Comparative evaluation of antifibrotic activity of Salvadora persica extracts in CCl4-induced liver fibrosis in rats by reducing oxidative stress and inflammation via transcriptional modulation associated with TGF-β1/Smad signaling pathway
Source: Front Pharmacol. 2026 Oct 8;17:1900981. doi: 10.3389/fphar.2026.1900981 (PMC13688013; doi:10.3389/fphar.2026.1900981)

Supplementary Figures (1-3) of Total ion chromatograms (TIC) of plant *Salvadora persica* exracts

The following are the Total ion chromatograms (TIC) of plant *Salvadora persica* (SP) extracts, aqueous (SP-AQ), ethanol (SP-E) and petroleum ether (SP-PE).

The chromatograms of SP extracts are labelled with nearly all phytochemicals that are given in manuscript Table 5. However, a few minor constituents could not be individually labeled because their signals originate from overlapping or unresolved peaks in the highly complex TICs, making their visual assignment impractical despite their successful identification through the deconvolution and spectral matching procedures.

Figure 1. TIC of SP-AQ extract

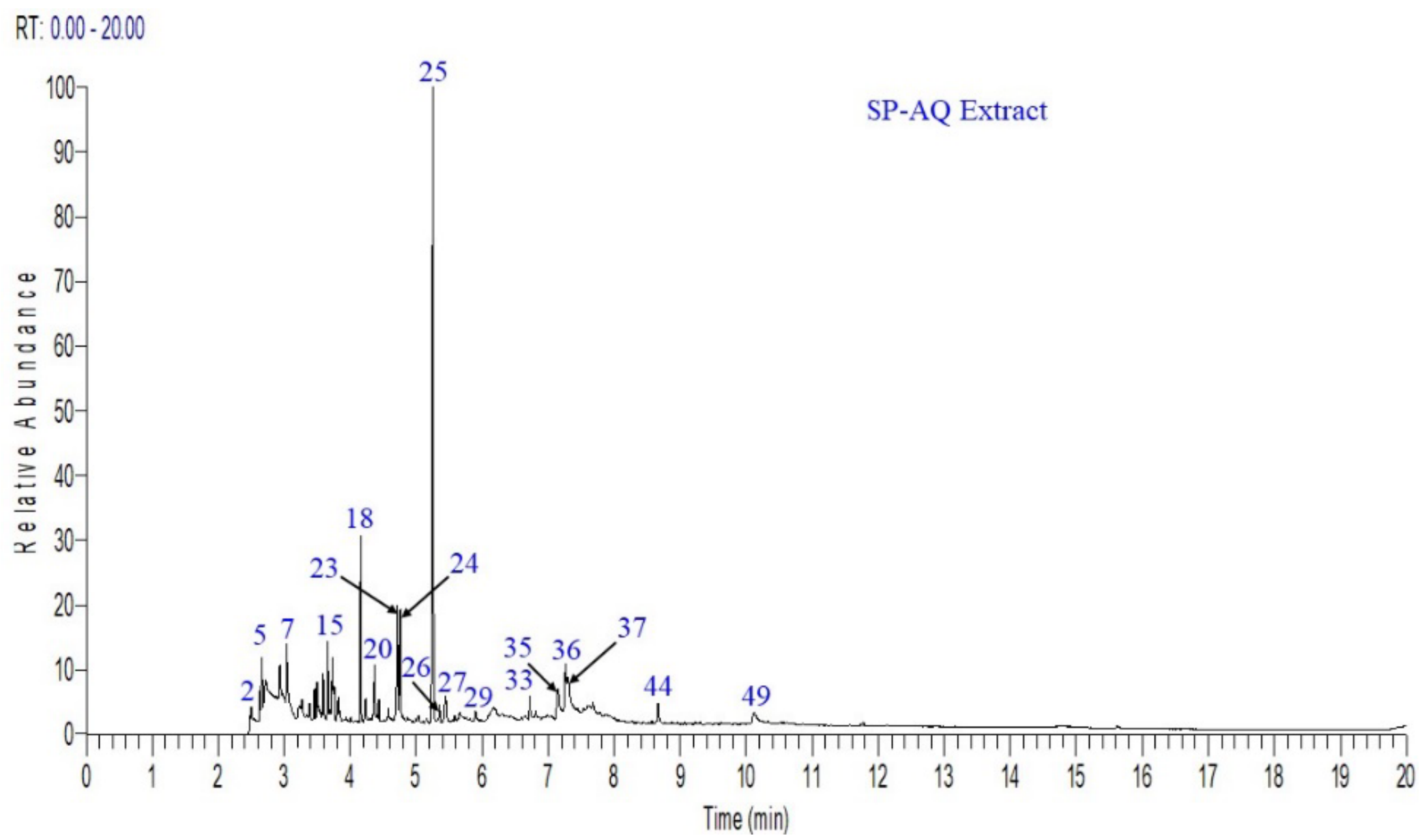

Figure 2. TIC of SP-E extract

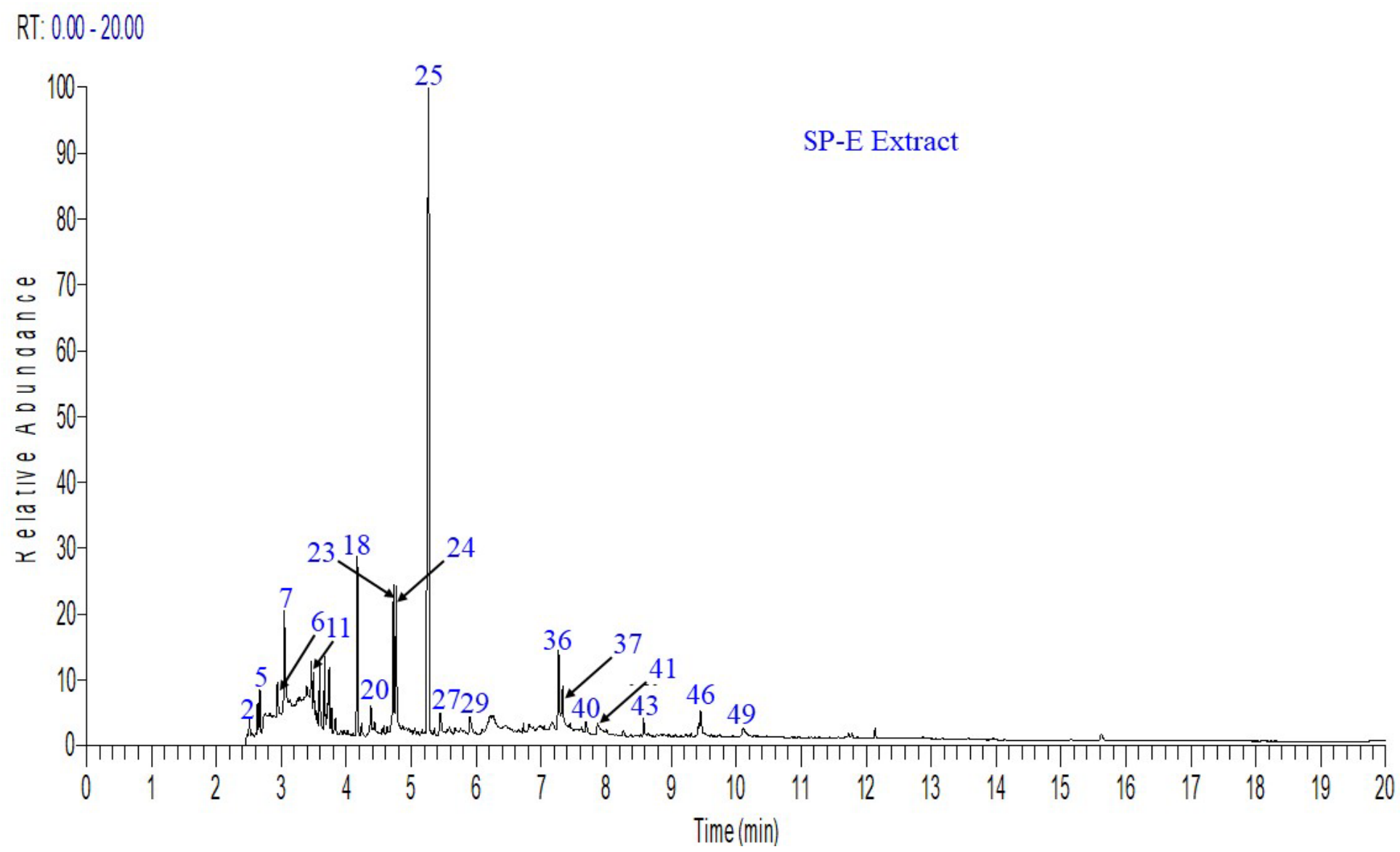

Figure 3. TIC of SP-PE extract

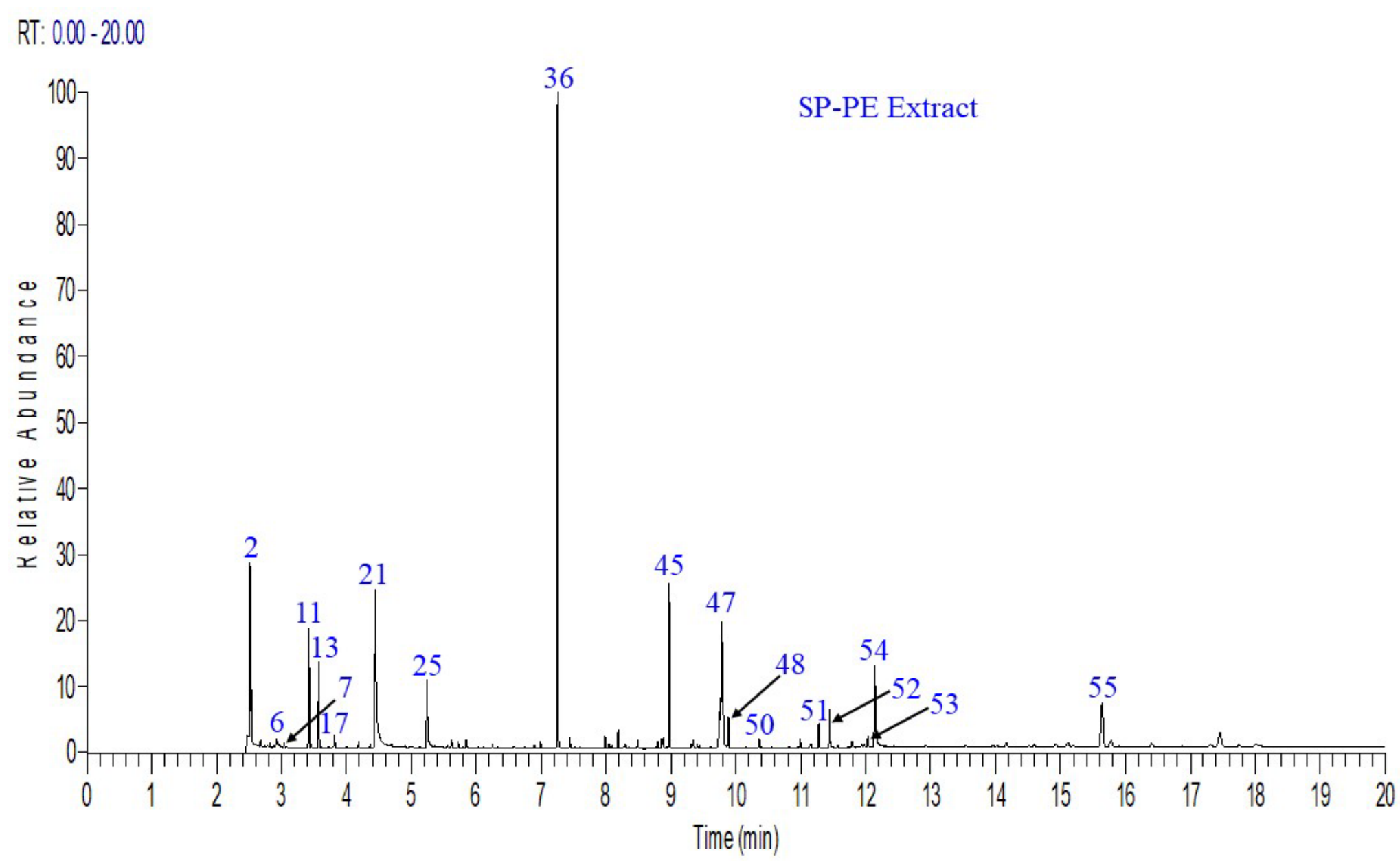

Supplement: Supplementary file 1 [file Image1.pdf]
